# Phospho*enol*pyruvate Carboxylase Identified as a Key Enzyme in Erythrocytic *Plasmodium falciparum* Carbon Metabolism

**DOI:** 10.1371/journal.ppat.1003876

**Published:** 2014-01-16

**Authors:** Janet Storm, Sonal Sethia, Gavin J. Blackburn, Achuthanunni Chokkathukalam, David G. Watson, Rainer Breitling, Graham H. Coombs, Sylke Müller

**Affiliations:** 1 Institute of Infection, Immunity & Inflammation, College of Medical, Veterinary and Life Sciences, University of Glasgow, Glasgow, United Kingdom; 2 Strathclyde Institute of Pharmacy and Biomedical Sciences; University of Strathclyde, Glasgow, United Kingdom; 3 Glasgow Polyomics, Wolfson Wohl Cancer Research Centre, University of Glasgow, Glasgow, United Kingdom; 4 Manchester Institute of Biotechnology, Faculty of Life Sciences, University of Manchester, Manchester, United Kingdom; National Institute for Medical Research, United Kingdom

## Abstract

Phospo*enol*pyruvate carboxylase (PEPC) is absent from humans but encoded in the *Plasmodium falciparum* genome, suggesting that PEPC has a parasite-specific function. To investigate its importance in *P. falciparum*, we generated a *pepc* null mutant (D10^Δ*pepc*^), which was only achievable when malate, a reduction product of oxaloacetate, was added to the growth medium. D10^Δ*pepc*^ had a severe growth defect *in vitro*, which was partially reversed by addition of malate or fumarate, suggesting that *pepc* may be essential *in vivo*. Targeted metabolomics using ^13^C-U-D-glucose and ^13^C-bicarbonate showed that the conversion of glycolytically-derived PEP into malate, fumarate, aspartate and citrate was abolished in D10^Δ*pepc*^ and that pentose phosphate pathway metabolites and glycerol 3-phosphate were present at increased levels. In contrast, metabolism of the carbon skeleton of ^13^C,^15^N-U-glutamine was similar in both parasite lines, although the flux was lower in D10^Δ*pepc*^; it also confirmed the operation of a complete forward TCA cycle in the wild type parasite. Overall, these data confirm the CO_2_ fixing activity of PEPC and suggest that it provides metabolites essential for TCA cycle anaplerosis and the maintenance of cytosolic and mitochondrial redox balance. Moreover, these findings imply that PEPC may be an exploitable target for future drug discovery.

## Author Summary

The genome of the human malaria parasite *Plasmodium falciparum* encodes a protein called phospho*enol*pyruvate carboxylase (PEPC) absent from the human host. PEPC is known to fix CO_2_ to generate metabolites used for energy metabolism in plants and bacteria, but its function in malaria parasites remained an enigma. Our study aimed to elucidate the role and importance of PEPC in *P. falciparum* in its host red blood cell by generating a gene deletion mutant in *P. falciparum*. This was only achievable in the presence of high concentrations of malate were added to the culture medium. The mutant generated (D10^Δ*pepc*^) had a severe growth defect, which was rescued partially by malate or fumarate (but not any other downstream metabolites), suggesting that they feed into the same metabolic pathway. Using heavy isotope labelled ^13^C-U-D-glucose and ^13^C-bicarbonate we showed that PECP has an important role in intermediary carbon metabolism and is vital for the maintenance of cytosolic and mitochondrial redox balance. Together these findings imply that PEPC may be an exploitable target for future drug discovery.

## Introduction

*Plasmodium falciparum* is the causative agent of malaria, a disease claiming an estimated 660,000 lives annually, primarily among children in Africa [1]. The spread of drug resistance and the lack of an efficacious vaccine hinder the control of the disease, and new targets for chemotherapies or vaccine development need to be identified and exploited [2]. The development of the parasite and its modification of the host's red blood cells (RBC) cause the pathogenesis of malaria, but the biochemical adaptations enabling this remain to be fully elucidated.

Energy generation in *P. falciparum* asexual erythrocytic stages depends upon glucose being primarily converted to lactate by anaerobic glycolysis, which is then excreted as the major metabolic end product [3]. This is consistent with the finding that in *P. falciparum* the pyruvate dehydrogenase complex (PDH) is solely present in a plastid-like organelle, the apicoplast, where it provides acetyl-CoA for fatty acid biosynthesis and possibly other acetylating reactions [4], [5]. It was shown very recently, however, that despite the absence of mitochondrial PDH, pyruvate can be metabolised by a PDH-like enzyme complex [6] and oxidised through a forward tricarboxylic acid (TCA) cycle in the erythrocytic stages of *P. falciparum*, being particularly important in the gametocytes, and also in the related apicomplexan parasite *Toxoplasma gondii* [3], [6], [7]. These findings are in contrast to previous reports that there is no link between cytosolic glucose catabolism and mitochondrial TCA metabolism [8], although this report was later retracted [9].

A striking feature of carbon metabolism in *Plasmodium* is their ability to fix CO_2_. This is utilised to generate carbamoyl phosphate and thence pyrimidines and also is incorporated into amino acids and α-ketoacids in *P. lophurae*, *P. knowlesi* and *P. berghei* [10]–[12]. Together with Trager's finding that *P. falciparum* require CO_2_ for *in vitro* growth [13], this suggests that CO_2_ fixation is necessary for the parasite's intra-erythrocytic survival. CO_2_ fixation may occur via carbamoyl phosphate synthase, phospho*enol*pyruvate carboxylase (PEPC) and/or phospho*enol*pyruvate carboxykinase (PEPCK), with the latter two feeding into intermediary carbon metabolism [14]–[16]. *P. falciparum* PEPCK is primarily expressed in gametocytes and mosquito stages [16], and is generally considered to produce phospho*enol*pyruvate (PEP) and release CO_2_. Thus PEPC, which utilises bicarbonate, generated from CO_2_ by carbonic anhydrase in *P. falciparum* [17], to convert PEP into oxaloacetate (OAA) is a strong candidate to have a key role in *P. falciparum* carbon metabolism by fixing CO_2_. Plant and bacterial PEPCs have been well characterised [18]–[20]; the malarial enzyme has, however, been little studied. There is just one report on *P. berghei* PEPC activity [14], even though PEPC is absent from mammals and thus potentially offers great opportunities for exploitation by novel antimalarial intervention strategies. Thus this study aimed to confirm the operation of PEPC in erythrocytic stages of *P. falciparum*, to elucidate its contributions to intermediary carbon metabolism and intra-erythrocytic survival, and so determine the likelihood that it is a viable drug target.

## Results

### Knockout of *pepc* gene by homologous recombination

**Figure 1:**
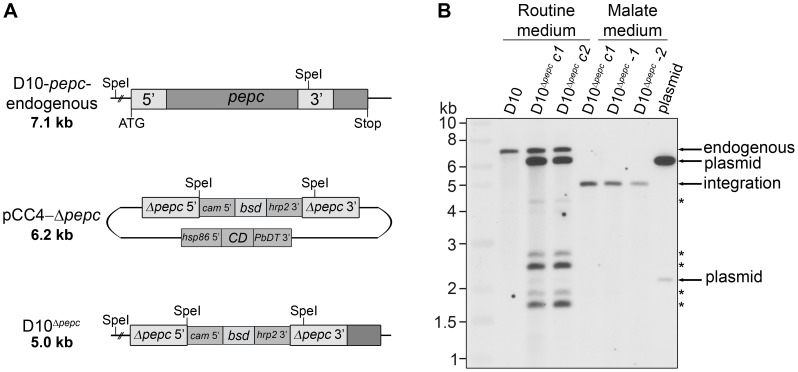
Gene replacement of *pepc*. (A) Schematic representation of the endogenous *pepc* locus, the pCC4-Δ*pepc* plasmid and the *pepc* locus following integration by double crossover recombination of pCC4-Δ*pepc* (D10^Δ*pepc*^). SpeI restriction sites for diagnostic Southern blot analyses and sizes of the expected DNA fragments are indicated. Two regions homologous to the 5′ (Δ*pepc* 5′) and 3′ (Δ*pepc* 3′) end of *pepc* present in pCC4-Δ*pepc* recombine with the endogenous *pepc* locus and part of the *pepc* gene is replaced with the positive selectable marker *blasticidin-S-deaminase* (*bsd*), which is under control of the *calmodulin* promoter (*cam* 5′) and the *histine rich protein 2* 3′ UTR (*hrp2* 3′). The plasmid contains the negative selectable marker *cytosine deaminase* (*CD*), which is under control of the *heat shock protein 86* promotor (*hsp86* 5′) and the *P. berghei dihydrofolate reductase* 3′ UTR (*PbDT* 3′), and is lost upon double crossover recombination. (B) Southern blot of SpeI-digested genomic DNA of wild type parasites and parasites transfected with pCC4-Δ*pepc*, cultured in routine medium or malate medium, probed with the 5′ end of *pepc*. Cycle 1 (c1) refers to the first parasites resistant to blasticidin and 5-fluorocytosine (5-FC), while cycle 2 (c2) are 5-FC-resistant parasites after 1 additional drug selection cycle. Integration of the plasmid only occurred when the transfectants were cultured in malate medium (diagnostic fragment: 5.0 kb). The derived clones D10^Δ*pepc*^-1 and D10^Δ*pepc*^-2 are shown in lane 5 and 6. In routine medium no integration was observed, only fragments corresponding to endogenous *pepc* (7.1 kb), plasmid (6.2 kb and 2.1 kb) and five fragments of unknown identity (*) were detected.

Initially, a disruption of the *pepc* gene was attempted using a single homologous recombination approach with the plasmids pHH1-Δ*pepc* and pHH1-3′*pepc*. The control construct pHH1-3′*pepc* targeted the *pepc* locus and replaced the 3′ region of the gene, while the pHH1-Δ*pepc* construct did not integrate into the correct gene locus, as shown by pulsed field gel electrophoresis (Fig. S1). These data revealed that the *pepc* locus is not refractory to recombination, but that a gene disruption was unsuccessful probably because the *pepc* gene is very important or essential for parasite survival. Parasites were then transfected with the plasmid pCC4-Δ*pepc*, in order to target the *pepc* gene locus by double homologous recombination [21]. The *pepc* locus was not targeted when parasites were cultured in routine medium (which does not contain added malate); however, addition of 5 mM malate to the medium (malate medium) allowed the replacement of the gene with the selectable marker, *blasticidin deaminase* (*bsd*). This was verified by Southern blot analyses (Fig. 1). The D10^Δ*pepc*^ mutants were cloned, and two independent clones D10^Δ*pepc*^-1 and D10^Δ*pepc*^-2 (Fig. 1B) were used for phenotype analyses. The data for the two clones were effectively the same, and so one set of data are presented forthwith and the line referred to as D10^Δ*pepc*^.

### The *pepc* gene is important for intra-erythrocytic survival of *P. falciparum*

**Figure 2:**
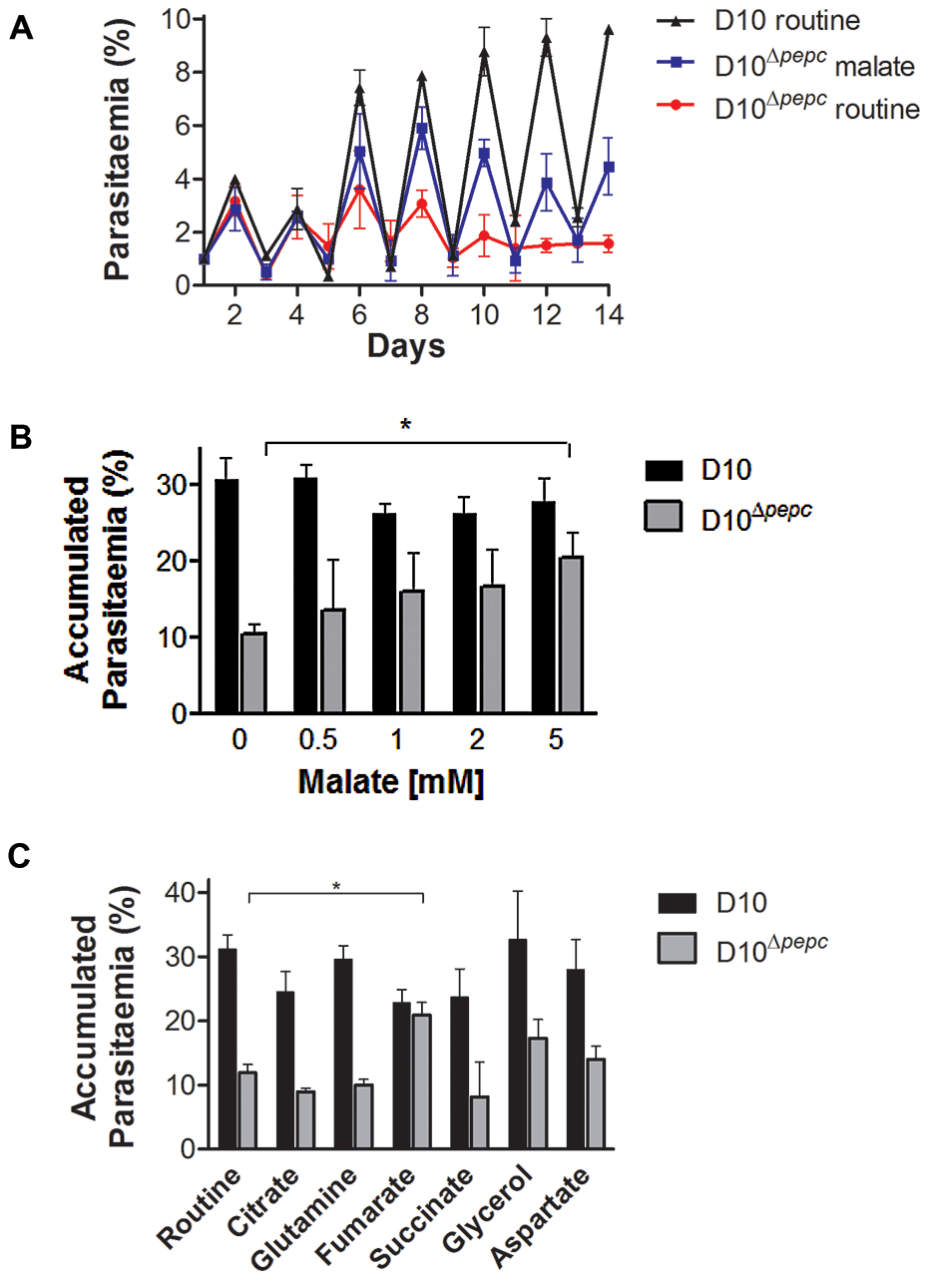
Growth phenotype of D10^Δ^ ^*pepc*^ and rescue by malate and fumarate. (A) Growth of D10 in routine medium lacking malate and D10^Δ*pepc*^ in malate or routine medium was followed for 14 days. The cultures were diluted 1∶5 on even days. The means ± S.D. of two (D10) and three (D10^Δ*pepc*^) replicates are shown. (B and C) D10 and D10^Δ*pepc*^ were cultured in routine medium for 9 days, synchronised, diluted to 1% parasitaemia and cultured for 5 days in medium supplemented with increasing concentrations of malate (0.5–5 mM) (B) or a range of metabolites (C). The cultures were diluted 1∶5 on day 3. The parasitaemia on day 5 was determined and multiplied with the dilution factor to give the accumulated parasitaemia. Data are means ± S.E.M. of at least 3 independent experiments, each done in triplicate (for succinate and citrate, means ± S.D. of 2 experiments done in triplicate). The asterisks indicate a statistically significant increase in parasitaemia compared to routine medium (p<0.05). Other apparent changes were not statistically significant. The final concentrations of metabolites used in (C) were: 0.5 mM citrate, 5 mM all others.

Given that the deletion of the *pepc* gene was achieved only when the culture medium was supplemented with 5 mM malate, the effect of withdrawing malate from the medium on the growth of D10^Δ*pepc*^ was analysed. Parasite growth in routine medium was followed for 14 days (Fig. 2A). After 6 days in routine medium, D10^Δ*pepc*^ had severely reduced growth rates and completely lost their synchronicity. Nevertheless, they continued to replicate a little, showing that in culture they are able to compensate to an extent for the loss of PEPC function. Likely mechanisms include obtaining some malate from the host erythrocyte directly or conversion from fumarate, generated as a by-product of purine salvage or itself taken up from the erythrocyte. The mutant parasites grew better in medium supplemented with malate, but the added 5 mM malate did not fully restore growth to the wild type rate (Fig. 2A). Lower concentrations of malate were less effective (Fig. 2B), whereas applying higher concentrations of malate did not improve growth further (data not shown). The beneficial effect of malate for growth of D10^Δ*pepc*^ seemed likely to be due to providing the mutant parasite with one of the downstream products of PEPC metabolism, and this post-PEPC metabolism is important for progression of *P. falciparum* through their erythrocytic cycle. In order to ascertain whether other possible downstream metabolites of PEPC could have beneficial effects, we tested several for growth stimulation of D10^Δ*pepc*^. The only metabolite other than malate that significantly restored parasite growth was fumarate (Fig. 2C). This suggests that supplementation of the medium with fumarate may also have allowed experimental deletion of the *pepc* gene similarly to malate supplementation, although this possibility was not tested. Notably glutamine was unable to rescue growth, probably a reflection of the parasite's metabolism of this amino acid as described below.

### Analysis of the *pepc* gene product

**Figure 3:**
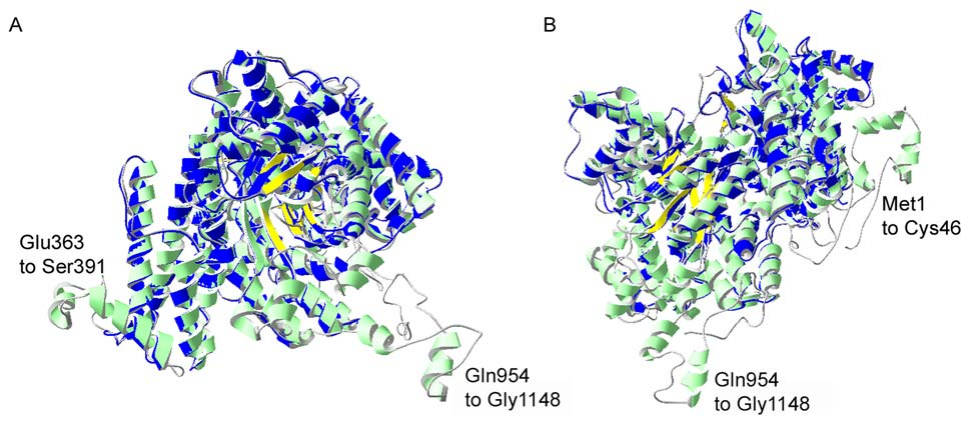
I-TASSER homology model of *P. falciparum* PEPC. Structural alignment of an I-TASSER predicted model for *Pf*PEPC with the top structural analogue *Ec*PEPC is shown. *Pf*PEPC is represented in a ribbon diagram where α-helices are in green, β-sheets are in yellow and coils are in grey. The *Ec*PEPC ribbon diagram is represented completely in blue. Coincident structures are dual-coloured. Based on the homology modelling, three regions of *Pf*PEPC were found not to overlap with *Ec*PEPC. Two of these regions are visible in (A), (Glu363 to Ser391 and Gln954 to Gly1148). The structure rotated to the left is displayed in (B) to show the third non-homologous region (Met1 to Cys46). The C-terminus of *Pf*PEPC appears to be entirely non-homologous with *Ec*PEPC presumably because of a long *Plasmodium-*specific insertion (from Gln954) that separates the terminal 9 amino acid sequence, which is highly conserved in the parasite protein, from the core structure (Fig. S2).

The predicted protein encoded by the *pepc* gene of *P. falciparum* has clear similarity with PEPCs of other organisms but rather limited identity. Amino acid identities between *P. falciparum* PEPC and plant enzymes are in the range of 30% (those of *Zea mays* and *Arabidopsis thaliana* are 29% and 31% identical, respectively) whereas identity with *Escherichia coli* PEPC is 25%. An extension at the N-terminus of the *P. falciparum* protein, a 29 amino acid central insertion and a long insertion towards the C-terminus (see alignment Fig. S2) are the prime reasons for this low degree of sequence identity and also the larger mass of the parasite protein (134 kDa compared with 100 kDa of bacterial PEPC and 116 kDa of plant PEPC) [18]. Nevertheless, the parasite protein does contain residues known to be crucial to the activities of other PEPCs. The C-terminal catalytic peptide at position 1143 to 1148 (equivalent to 966 to 970 of maize PEPC) (see Fig. S2) is completely conserved in *P. falciparum* PEPC as are residues R685, which is involved in PEP binding (equivalent to R647 in maize PEPC), and H193 (equivalent to H177 in maize PEPC), which acts as catalytic base in the active site of the protein [18]. In addition, the two loops probably involved in regulation of PEPC catalytic activity (loop I and loop II in Fig. S2) are also highly conserved in *P. falciparum* PEPC. Loop I comprising residues ^678^GRGGXXG^685^RGG (XX = SV in *P. falciparum* PEPC;  = TV in maize PEPC) includes the active site residue R685 and allows flexibility of this residue during the catalytic cycle. It is suggested that in the maize enzyme when in its inactive state the equivalent arginine residue is ‘attracted away’ from the active site, whereas in the active state R685 is attracted to the active cavity through interaction with the C-terminal glycine. Loop II is located at the catalytic cavity and forms a bridge above the β-barrel; it is thought to be involved in binding of HCO_3_^−^ and may have a role in covering the active site cavity to protect catalytic intermediates from unwanted reactions with surrounding water [18]. Moreover, a homology model of *P. falciparum* PEPC that was generated using ITASSER (Fig. 3) is consistent with it having a good degree of structural similarity with *E. coli* PEPC. This shows that the core structure and the location of catalytically and regulatory amino acids in *P. falciparum* PEPC are structurally in good agreement with the equivalent in the PEPC of *E. coli*, with the β-barrel that generates the active site cavity being structurally conserved and similarly surrounded by α-helices. The homology model also shows that the N-terminus of *P. falciparum* PEPC forms a non-homologous loop (residues Met1 to Cys46 in Fig. 3). The two other insertion regions also show clear differences in structure from the *E. coli* PEPC, both being presented as surface-located loops (Fig. 3).

A full phylogenetic analysis of PEPCs was reported last year [22] and this suggests that *P. falciparum* PEPC is aligned with the C4 plant PEPCs as it possesses the signature amino acids K373 and S818; these are equivalent to K353 and S780 in the maize PEPC sequence (Fig. S2), are invariably found in C4 plant PEPCs and are thought to influence kinetic parameters and inhibition/activation features of the enzymes. In contrast, these residues in C3 plant PEPCs are arginine and alanine, respectively [23]. Scrutiny of other amino acid residues important for the protein's catalytic activity and binding of allosteric activators or inhibitors [18], [23], [24] are consistent with this conclusion. Interestingly, however, *P. falciparum* PEPC does not possess the N-terminal serine residue present in plant PEPC proteins that is phosphorylated in order to regulate PEPC activity of C4 plants [19]. However, analyses of the phosphoproteome of *P. falciparum* suggests that S1109 is phosphorylated during blood stage development of the parasites [25] which implies that *P. falciparum* PEPC activity may be regulated through phosphorylation of this residue.

The conservation of all of these features in the *P. falciparum* PEPC suggests that the gene encodes a functional PEPC protein. Unfortunately detailed biochemical and structural analyses of *Plasmodium* PEPC recombinant protein have so far been ruled out by our lack of success in attempts to express parasite protein in enzymatically active form; despite very extensive attempts using different expression systems and modified gene constructs (with the long insertions deleted), we have been unable to generate active recombinant enzyme that could be studied in a meaningful way. The native enzyme has been purified from *P. berghei* and displayed a molecular size of 280 kDa [14], implying that it forms a homodimer. Other larger PEPC species were also found after sucrose gradient centrifugation, suggesting that similar to PEPC isolated from other organisms the parasite enzyme may also form a tetramer [18]. Such analyses of the PEPC of *P. falciparum* have not been reported, obtaining sufficient pure enzyme has been problematic.

### Sensitivity of D10^Δ*pepc*^ to metabolic inhibitors

**Figure 4:**
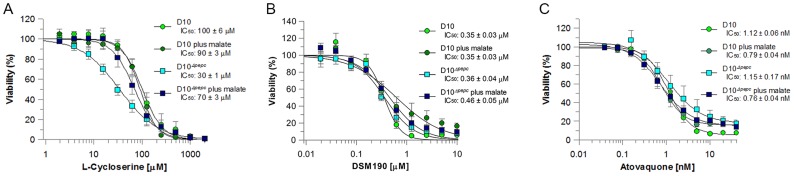
Efficacy of L-cycloserine, DSM190 and atovaquone against D10 and the D10^Δ^ ^*pepc*^ in routine and malate media. The effect of L-cycloserine (A), DSM190 (B) and atovaquone (C) on parasite viability. D10^Δ*pepc*^ was maintained for 9 days in routine medium prior to the experiment.

To probe the mechanism facilitating the survival of D10^Δ*pepc*^, its susceptibility to metabolic inhibitors was quantified. L-cycloserine, a competitive inhibitor of pyridoxal phosphate-dependent enzymes such as aspartate aminotransferase (AAT) [26], showed a clear differential effect on the parasite lines. D10^Δ*pepc*^ in routine medium was more sensitive (IC_50_ of 30±1 µM compared to 100±6 µM for D10, p<0.05; Fig. 4A), an effect partially reversed when D10^Δ*pepc*^ was cultured in malate supplemented medium (IC_50_ of 70±3 µM). We analysed by western blotting the expression levels of both AAT and malate dehydrogenase (MDH), enzymes important for metabolism of OAA generated by PEPC, and showed that they were not significantly affected by the deletion of the *pepc* gene regardless of the presence or absence of malate in the culture medium (Fig. S3). These data are entirely consistent with D10^Δ*pepc*^ depending on the activity of aminotransferases such as AAT and being more sensitive to the competitive inhibitor as result of lower OAA levels in the absence of PEPC.

AAT generates aspartate, which is a precursor for pyrimidine synthesis. Therefore the parasites' sensitivity to DSM190, an inhibitor of the key enzyme of pyrimidine biosynthesis dihydroorotate dehydrogenase (DHOD) [27], was determined and it was found that D10 and D10^Δ*pepc*^ were equally susceptible to the inhibitor, regardless of the presence of malate (Fig. 4B), suggesting that DHOD function is not affected in D10^Δ*pepc*^. In the mitochondrion, malate is converted to OAA by malate∶quinone oxidoreducatase (MQO) which transfers electrons to the mitochondrial electron transport chain (mtETC) [28] and so we hypothesised that mtETC could be compromised by the absence of PEPC. To address whether this was the case, the sensitivities to atovaquone, an inhibitor acting against cytochrome *bc1* complex in the mtETC [29], [30], were determined for D10 and D10^Δ*pepc*^ in routine or malate medium. Atovaquone inhibited the parasite lines with similar IC_50_ values between 0.7 and 1.2 nM (Fig. 4C). We also compared the mitochondrial membrane potential of D10 and D10^Δ*pepc*^ parasites by monitoring the uptake of the fluorophore MitoTracker. The data acquired indicate that the electrochemical gradient across the inner mitochondrial membrane is not affected by the loss of PEPC function (Fig. S4). To confirm the validity of this approach, the mitochondrial membrane potential was collapsed with valinomycin; this resulted in an even distribution of the fluorophore throughout the cytosol of the parasite.

### Metabolomic analysis of D10^Δ*pepc*^

The data above are consistent with PEPC generating OAA through CO_2_ fixation with subsequent conversion into malate and aspartate, and that these pathways are important for parasite development. That both malate and fumarate restore the growth phenotype implies that they feed into the same pathways. Intermediary metabolism is, however, a complicated matrix of interactions and the exogenously supplied malate and fumarate could provide their beneficial effects via conversion to a number of metabolites including OAA itself. So we decided that an analysis of key metabolites in this part of metabolism would inform in more detail on the contributions of PEPC. Thus a targeted metabolomics analysis using liquid chromatography-mass spectrometry (LC-MS) with the stable-isotope labelled nutrients ^13^C-U-D-glucose, ^13^C-bicarbonate and ^13^C,^15^N-U-glutamine was conducted.

One difficulty in performing these labelling experiments was that the growth defect of the mutant parasites restricted the experiments that could be done. Whereas wild type D10 parasites can be synchronised and maintained for the 28-hour incubation period with the stable isotope at up to 10% parasitaemia, the D10^Δ*pepc*^ mutant grown without added malate for 9 days only reached a parasitaemia of 6%, maximally. In addition, these parasites could not be concentrated efficiently using MACS columns in the same way as the D10 parasites. The explanation for this is unclear, but perhaps could reflect some disruption of haemozoin synthesis in the mutant parasites. To overcome these difficulties, we carried out direct comparisons of D10^Δ*pepc*^- and D10-infected RBC at 6% parasitaemia and, to ensure that the data from these experiments were robust, we also analysed D10 parasites incubated under the same conditions but subsequently concentrated to 90% parasitaemia via the MACS column methodology [31].

**Figure 5:**
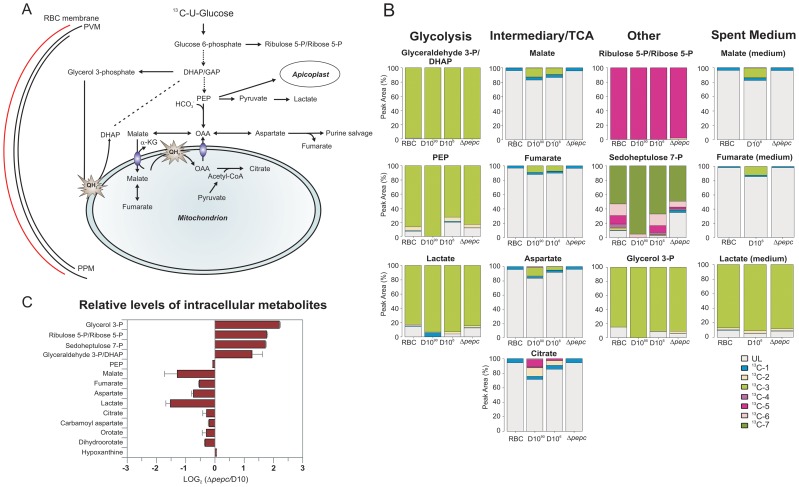
Metabolite labelling from ^13^C-U-D-glucose. (A) Schematic representing glucose utilisation in *P. falciparum* based on the utilisation of ^13^C-U-D-glucose and distribution of ^13^C carbons into metabolic intermediates. (B) The parasites perform glycolysis such that triose 3-phosphates, phospho*enol*pyruvate (PEP) and lactate had very extensive labelling with ^13^C from ^13^C-U-D-glucose (all are three carbon compounds, hence exist mainly as ^13^C-3 molecules as rapidly generated from glucose by glycolysis). ^13^C is also incorporated into malate, fumarate and aspartate through the action of PEPC (and hence the ^13^C-3 molecules) as well as the TCA metabolite citrate by the action of a PDH-like enzyme [3], [6] in D10 parasites. Citrate is formed from OAA (occurring as ^13^C-3 molecule as well as unlabelled) and acetyl-CoA (presumably mainly ^13^C-2 molecules as generated from pyruvate) and so was present as ^13^C-2 and ^13^C-5 molecules as well as unlabelled. ^13^C-labelled malate, fumarate, asparate and citrate were absent from samples of D10^Δ*pepc*^ (Δ*pepc*). ^13^C-U-D-glucose was also fed into the PPP intermediates ribulose 5-P/ribose 5-P and sedoheptulose 7-P (compounds with 5 and 7 carbons, respectively, hence the extensive presence as ^13^C-5 and ^13^C-7 molecules) and used to generate glycerol 3-phosphate (also a 3-carbon molecule hence the ^13^C-3 labelling). Legend: UL, unlabelled; ^13^C-1: one carbon atom labelled; ^13^C-2 to ^13^C-7: two to seven carbon atoms labelled; D10^90^, D10-infected RBC concentrated to 90% parasitaemia; D10^6^, D10-infected RBC at 6% parasitaemia. (C) Relative levels of intracellular metabolites in D10^Δ*pepc*^ (Δ*pepc*) and D10. Abbreviations: α-KG, α-ketoglutarate; DHAP, dihydroxyacetone phosphate; GAP, glyceraldehyde 3-phosphate; OAA, oxaloacetate; PEP, phospho*enol*pyruvate; PPM, parasite plasma membrane; PVM, parasitophorous vacuole membrane; QH_2_, ubiquinol.

Incubation of parasites with ^13^C-U-D-glucose led, as expected, to an efficient incorporation of the ^13^C-labelled carbon skeleton into glycolytic intermediates and the end product lactate, both intracellularly and in the spent medium (Fig. 5, Table S1). The pentose phosphate pathway (PPP) metabolites ribulose 5-phosphate/ribose 5-phosphate and sedoheptulose 7-phosphate were also heavily labelled and apparently more abundant in D10^Δ*pepc*^ compared with D10 (Figs. 5B, 5C), providing evidence that the PPP is operational. In addition, levels of glycerol 3-phosphate were also greatly increased in D10^Δ*pepc*^ and strongly labelled (Figs. 5B, 5C). The proportion of incorporation of ^13^C label into most of these metabolites was not apparently different in D10^Δ*pepc*^ compared with D10, the only clear difference observed was reduced incorporation of ^13^C label into sedoheptulose 7-phosphate. Analysis of metabolites in the spent media of the samples (Table S1) indicated a large reduction of lactate production in D10^Δ*pepc*^, reflecting a similar reduction in glucose utilisation, signifying that the mutant parasites have a lower metabolic capacity than D10. To confirm this and quantify the relative generation of metabolic end-products and utilisation of substrates fully (mass spectrometric analysis is only semi-quantitative and is usually not used to quantify metabolite concentrations, although it can give an indication), we determined the concentrations of various metabolites in the spent media of the different parasite lines using enzyme-based assays. The results (Table 1) show that both the consumption of glucose and release of lactate are reduced to approximately half in the D10^Δ*pepc*^, and that lactate apparently accounted for approximately 75% and 70% of the glucose consumed by D10 and D10^Δ*pepc*^, respectively. The slightly lower relative generation of lactate by D10^Δ*pepc*^ is consistent with some glucose being diverted into pathways other than only glycolysis, such as the PPP and glycerol 3-phosphate production (see Fig. 5).

**Table 1:** Metabolite concentrations in spent medium.

Metabolite	RBC	D10	D10^Δ*pepc*^
Glucose (mM)	9.35±0.40	7.32±0.36	8.38±0.16*
Lactate (mM)	0.36±0.05	3.47±0.29	1.74±0.26**
Glutamine (mM)	1.50±0.13	0.96±0.11	1.52±0.11**
Succinate (mM)	<0.006	<0.006	<0.006
Malate (mM)	<0.005	<0.005	<0.005
Fumarate (mM)	<0.033	<0.033	<0.033

All data are means of three independent experiments with standard deviations.

: Glucose utilisation was significantly lower in D10^Δ*pepc*^ compared to D10 (p<0.05).

: Lactate excretion and glutamine utilisation were significantly lower in D10^Δ*pepc*^ compared to D10 (p<0.001). Succinate, malate and fumarate concentrations in the spent medium were below the detection limit of the assay kits used.

D10 parasites also incorporated ^13^C label from glucose into intracellular ^13^C-3-labelled malate, fumarate and aspartate, the first two also being released into the medium, which can be explained by ^13^C-3-labelled PEP being utilised by PEPC to fix CO_2_ and form OAA (Fig. 5B). Most importantly, this was confirmed by the finding that these metabolites were not labelled in D10^Δ*pepc*^, validating the activity of PEPC in generating them. The presence of ^13^C in these metabolites was relatively greater in the samples extracted from D10-infected RBC concentrated to 90% compared to those with 6% parasitaemia, whereas no ^13^C label was detected in the RBC control (Fig. 5B), thus substantiating that the incorporation was parasite-specific. The total levels of malate, fumarate and aspartate were lower in D10^Δ*pepc*^ compared to D10-infected RBC, in contrast to glycerol 3-phosphate and the PPP metabolites (Fig. 5C). The release of malate and fumarate into the spent medium by D10 parasites, although detected by the mass spectrometric analysis, was less than we could measure using our enzyme-based assays (5 and 33 µM, respectively) (Table 1), emphasising that these metabolites are important in intermediary carbon metabolism in the parasite rather than being metabolic end-products (they could account for <0.5% and <2% of the glucose used, maximally).

Interestingly, we also detected ^13^C-2- and ^13^C-5- labelled citrate in D10, strongly supporting the presence of a canonical TCA metabolism operating in the erythrocytic stages of *P. falciparum* at least as far as citrate. Citrate was not ^13^C-labelled in D10^Δ*pepc*^, thus demonstrating that PEPC is a key node in intermediary carbon metabolism and forms a crucial link between glycolysis and mitochondrial TCA metabolism.

**Figure 6:**
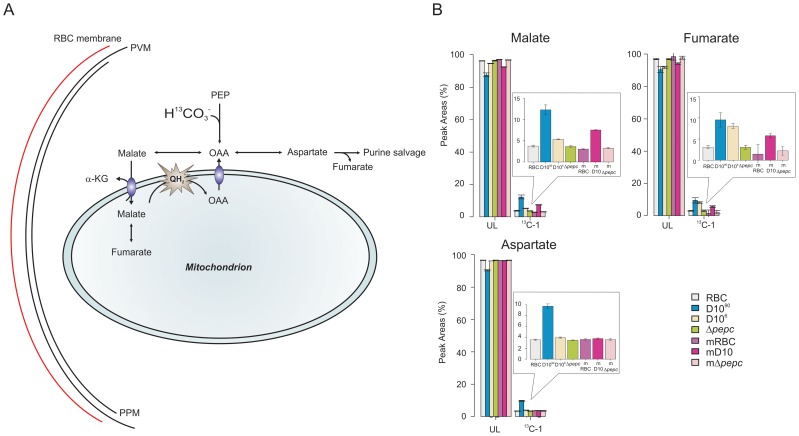
Metabolite labelling from ^13^C-bicarbonate. (A) Schematic representing bicarbonate utilisation in *P. falciparum* by determining the incorporation of the single ^13^C isotope into metabolic intermediates. (B) ^13^C-bicarbonate is incorporated into malate and fumarate and also to a lesser extent into aspartate and citrate. The natural relative abundance of ^13^C-1 label in metabolites was not subtracted from the data shown. This accounts for ∼1.1% of ^13^C-1 for each carbon of the metabolites in all samples analysed including those of D10^Δ*pepc*^ (Δ*pepc*) and RBC, which show no specific additional incorporation of ^13^C-bicarbonate into their carbon skeleton. Thus the specific incorporation into the metabolites is most usefully assessed by comparing the levels in parasite-infected cells with that measured in the RBC samples (which reflects the natural abundance of the isotope). Abbreviations: as in Figure 5 and m, spent medium samples.

These findings using ^13^C-U-D-glucose were further validated by the analyses of parasites incubated with ^13^C-HCO_3_^−^ (Fig. 6, Tables S3 and S4). The single ^13^C carbon present in bicarbonate was detected in malate, fumarate and aspartate in D10, with generally higher levels of incorporation detected in the samples extracted from infected RBC with 90% parasitaemia. Importantly, the incorporation of ^13^C label was completely absent in D10^Δ*pepc*^-infected RBC (the ^13^C label present in these samples was at a similar level to that in the RBC control and attributed to natural occurrence of ^13^C-1 isotopomers), confirming that CO_2_ fixation into these metabolites is indeed attributable to PEPC. Overall these data provide clear evidence that PEPC functions in CO_2_ fixation and has an anaplerotic role.

We also investigated the catabolism of ^13^C ^15^N-U-glutamine by the parasites. The data obtained (Fig. S5 and Tables S5 and S6,) showed that there was a good flux from glutamine, via glutamate, to α-ketoglutarate and subsequently succinate, which were present as ^13^C-5- and ^13^C-4-labelled molecules, respectively, in high proportion. The amount of ^13^C-4-labelled succinate in the D10^Δ*pepc*^ parasites was less than in D10, reflecting the lower catabolic rate generally in the mutant and also the lower consumption of glutamine (Table 1). There was, however, very little ^13^C label detected in the malate and fumarate pools. This is consistent with the conversion of succinate to these metabolites being rather low as also described by others recently [6], although the dilution effect of unlabelled forms of these metabolites in the host erythrocytes is also a factor. This may also explain why added glutamine was unable to restore growth of D10^Δ*pepc*^, unlike malate and fumarate (Fig. 2), although the mitochondrial location of the metabolites generated from glutamine may also be an important factor.

These data for glutamine metabolism together with those presented in Figs. 5 and 6 for metabolism of glucose and bicarbonate suggest that there is a full forward TCA cycle operating in the parasites. However, there appear to be three separate segments of the TCA pathway which are apparently fed by separate ‘entry points’ (malate, pyruvate and α-ketoglutarate, respectively). The fluxes through the reactions from malate and pyruvate to citrate as well as that from α-ketoglutarate to succinate seem to be greater than the flux through the other segments of the cycle. This could be due to citrate and succinate being removed from the cycle and fed into other metabolic activities, indeed citrate exiting the mitochondrion could be part of the parasite's malate shuttle as discussed below.

Given the complex matrix of interconnections between different areas of metabolism, it was conceivable that the deletion of *pepc* would impact upon parts of metabolism in addition to redox homeostasis and the TCA cycle and that these effects could be contributing to the growth defect. Purine and pyrimidine metabolism were candidate areas. Thus we analysed the mass spectrometry data to find whether or not the levels of several metabolites in this metabolic area are changed in the mutant parasites. The results (Table S7, Fig. 5C) suggest that there are relatively minor changes to carbamoyl aspartate, orotate, dihydroorotate and hypoxanthine, indicating that these pathways are not greatly affected by the gene deletion.

### PEPC as a possible drug target

The lack of PEPC in mammals and the importance of this enzyme to *P. falciparum*, as this study confirms, makes the parasite enzyme a possible drug target of interest. Plant and bacterial PEPCs are inhibited by 3,3-dichloro-2-(dihydroxyphosphinoylmethyl) propenoate (DCDP), an analogue of PEP. DCDP has a strong inhibitory effect on photosynthetic activity of plants at 1 mM and inhibits PEPC enzyme activity in the µM range [32]. The lack of recombinant protein has negated testing the effect of this PEP analog on the activity of *P. falciparum* PEPC, but we have assessed the efficacy of the inhibitor against parasite growth *in vitro*. The IC_50_ value for the inhibition of D10 was found to be 10.6±0.9 mM (n = 5); given this high concentration required to achieve a lethal effect on wild type parasites we did not test this compound against the mutant line. This poor efficacy of DCDP is likely to be due to its charged nature and thus to its inability to penetrate the parasite membranes.

## Discussion

Early studies with various *Plasmodium* species showed that bicarbonate incorporation into intermediary metabolites potentially involved TCA activity, suggesting a link between CO_2_ fixation and TCA anaplerosis in these parasites [10]–[12]. However, subsequent analyses revealed that *Plasmodium* species lack typical anaplerotic enzymes such as pyruvate carboxylase and malic enzyme (providing OAA and malate, respectively, to the TCA cycle in other organisms), and also mitochondrial aspartate∶glutamate antiporter and aminotransferase [33]–[35], all integral to the malate shuttle in mammalian cells. Moreover, it was reported and apparently accepted dogma that there was no link between cytosolic glucose catabolism and mitochondrial TCA metabolism [8], [36], although the original paper has been recently retracted [9]. Therefore, to reconcile these reports we postulated and tested the hypothesis that PEPC, a CO_2_ fixing enzyme usually found in plants and bacteria, performs anaplerosis in erythrocytic *P. falciparum* by maintaining a mitochondrial malate shuttle and thereby performs an important metabolic function essential for the growth of the parasite.

Our demonstration that the generation of D10^Δ*pepc*^ null mutants was only possible in the presence of mM concentrations of malate provides strong evidence that PEPC is indeed a critical enzyme for providing malate via OAA for important metabolic reactions. The severe growth defect of D10^Δ*pepc*^ in the absence of malate and the partial rescue upon malate re-supplementation support this conclusion. D10^Δ*pepc*^ survival in the absence of added malate or fumarate is presumably mediated through salvage of these metabolites from the host erythrocyte, although clearly the availability is insufficient to rescue the severely limited growth of D10^Δ*pepc*^ in culture (Fig. 2A). These data suggest that PEPC could be essential for *in vivo* growth and viability of the parasites and so potentially it is a valid drug target. Efforts have been made to target PEPC in plants for development of a novel herbicide [32], [37] and our results suggest that such inhibitors are worth considering for their antimalarial potential. We have tested a commonly known PEPC inhibitor for its ability to inhibit *P. falciparum* D10 viability and indeed this was possible, although only at very high concentrations. This poor efficacy is likely to be a reflection of the charged nature of the compound and it would be interesting, therefore, to assess in future studies the efficacy against *P. falciparum* of uncharged PEP analogues similar to those previously described [37], [38].

The availability of D10^Δ*pepc*^ has allowed us to investigate in detail the contributions that the enzyme makes to intermediary metabolism in the erythrocytic stages of the parasite. Using only samples at 6% parasitaemia means that a large component of the metabolites detected were from the erythrocytes themselves, which effectively dilutes out labelling of the same metabolites present in the parasites. Thus, we validated the data that we obtained with low parasitaemia cultures by concentrating labelled D10-infected RBC using the MACS procedure [31] and this reassuringly provided similar data. More detailed analysis of intermediary metabolism will require development of methodology allowing the analysis of the D10^Δ*pepc*^ null mutants at higher parasitaemia.

The targeted metabolomics analysis of the parasite-infected RBC, uninfected RBC and spent culture medium after incubation with ^13^C-U-D-glucose and ^13^C-bicarbonate has provided definitive evidence that PEPC produces OAA for the generation of malate, fumarate and aspartate and feeds into mitochondrial TCA metabolism. The proportion of the metabolites malate, fumarate and aspartate ^13^C-labelled in the D10-infected RBC was maximally <20%, even in the samples obtained from D10-infected RBC concentrated to ∼90% parasitaemia. This contrasts to the glycolytic and PPP intermediates and lactate that were extensively labelled from ^13^C-U-D-glucose. There are several contributory factors for this. Other substrates could feed into the PEPC pathway, such as fumarate from purine salvage using aspartate derived from haemoglobin digestion, and also OAA generated by PEPC being converted to aspartate via AAT – the idea of the latter being important is supported by the differential inhibition of D10 and D10^Δ*pepc*^ by L-cycloserine. The involvement of fumarate in post-PEPC metabolism is further supported by its ability to partially rescue the growth defect of D10^Δ*pepc*^ (Fig. 2), which also suggests that fumarate might be salvaged from the host cell, and is in line with the finding that radiolabelled fumarate is converted into malate, aspartate and OAA in isolated parasites [39]. Similarly, exogenous malate clearly can be used and is probably salvaged from the host erythrocyte, which could account for the survival of D10^Δ*pepc*^ in the absence of malate supplementation. Malate and fumarate are known to be interconverted in erythrocytes [40], [41], thus either of them may be the main metabolite salvaged, and it is probable that they are also interconverted by fumarase in the mitochondrion as this is generally a reversible reaction. In addition, glutamine is another substrate feeding into mitochondrial TCA metabolism, as shown here and previously by MacRae and colleagues and Cobbold and colleagues for *P. falciparum* and also *T. gondii* [3], [6], [7], leading to generation of (unlabelled) fumarate and malate. Importantly, however, the relatively high amounts of these metabolites in the RBC themselves are all unlabelled (Fig. 5, Table S1) and effectively dilute the labelled proportion of these metabolites detected in infected RBC, even in the concentrated samples of D10-infected RBC (Table S2). The detection of ^13^C- malate and ^13^C-fumarate in the spent medium of the ^13^C -U-glucose and ^13^C-bicarbonate experiments with D10 parasites, and the absence of this from the spent medium of D10^Δ*pepc*^ and RBC, also strongly supports the proposed activity of PEPC (Tables S1, S3). The explanation for the release of these metabolites requires further analysis, although our quantitative data on metabolites in the spent media show clearly not only that D10^Δ*pepc*^ has a catabolic deficiency reflecting its growth defect but also that in both D10 and D10^Δ*pepc*^ the major route of glucose catabolism is to lactate and there is very little release of succinate, malate or fumarate (Table 1). Overall, the data suggest that, as expected, there is a complicated interplay in intermediary metabolism between the parasite and its host cell.

During asexual erythrocytic life, *P. falciparum* generates ATP through glycolysis and primarily yields lactate as metabolic end-product [36] and our data confirm this (Fig. 5; Table S1). Importantly, our study corroborates the recent finding [3], [6] that an oxidative TCA pathway is also operational. The presence of the ^13^C-2-labelled citrate suggests that acetyl-CoA is used in the production of citrate in the mitochondrion, despite the lack of a mitochondrial PDH [4] which in most eukaryotes catalyses acetyl-CoA production in mitochondria, and supports the recent suggestion that instead branched chain α-ketoacid dehydrogenase acts to effect the conversion [3], [6]. The additional occurrence of ^13^C-5-labelled citrate, which is absent in D10^Δ*pepc*^, clearly supports malate being an additional entry point for mitochondrial TCA metabolism. This entry of malate into mitochondrial metabolism also allows for the transfer of reducing equivalents into the mtETC via MQO (Fig. 5).

**Figure 7:**
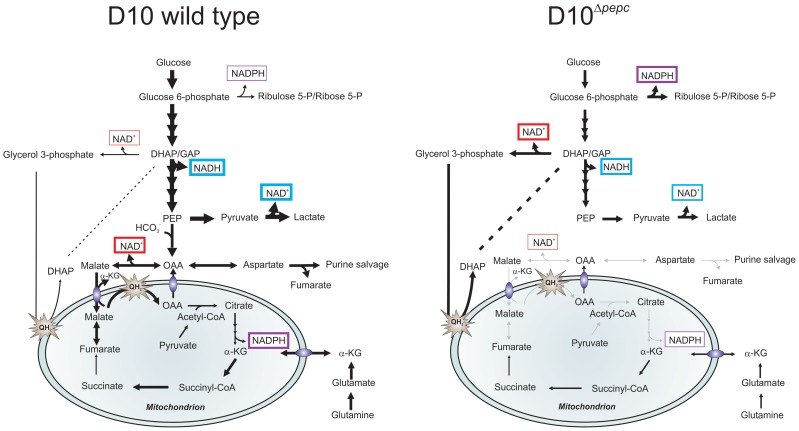
Hypothetical model of the contributions PEPC and adaptations of D10^Δ^ ^*pepc*^ to compensate for lack of PEPC. The width of arrows is indicative of the fluxes through the pathways. Glucose used by wild type parasites is phosphorylated into glucose 6-phosphate, which is either catabolised in the glycolytic pathway to generate lactate or is used in the PPP to provide NADPH and 5 and 7-carbon sugars for downstream metabolic processes. During glycolysis, NAD^+^ reduction and NADH oxidation (blue boxes) are balanced in the complete glycolytic sequence itself, but some metabolites are used in other ways. Glycerol 3-phosphate dehydrogenase and especially MDH, converting OAA (the product of CO_2_ fixation by PEPC) to malate, play roles in maintenance of redox balance in the cytoplasm (red boxes). Pyruvate is metabolised not only into lactate but is also transferred into the mitochondrion where it is oxidised into acetyl-CoA by a PDH-like dehydrogenase [3], [6]. Malate is transferred into the mitochondrion via a malate∶α-ketoglutarate (αKG) antiporter and enters TCA metabolism leading to OAA formation in the organelle, this oxidation transfers reducing equivalents into the mtETC (QH_2_). OAA may leave the mitochondrion, thus completing a parasite-specific form of a malate shuttle, or be converted to citrate as part of canonical TCA metabolism. Deletion of *pepc* leads to an imbalance in redox metabolism in both cytosol and presumably also mitochondrion. D10^Δ*pepc*^ apparently adapted to this with increased generation of glycerol 3-phosphate with concomitant NADH oxidation, thus compensating partially for the loss of downstream generation of NAD^+^ through MDH. Increased flux through oxidative PPP is a mechanism for generating NADPH which may be compensating for the reduced flux through TCA metabolism and therefore a reduced generation of NADPH at the IDH step in D10^Δ*pepc*^(purple boxes). The reduction in flux to lactate in D10^Δ*pepc*^ reflects the severely reduced growth phenotype of D10^Δ*pepc*^. The flux from glutamine via glutamate to α-ketoglutarate and, within the mitochondrion, to succinate and, at an apparently lower flux, to fumarate and malate is shown, with α-ketoglutarate as an additional entry point into TCA metabolism. The flux through these metabolites is lower in D10^Δ*pepc*^ compared with D10, reflecting the general growth defect of D10^Δ*pepc*^ and also its reduced consumption of glutamine, although the pathway in which the carbon skeleton is incorporated is not dramatically affected.

The labelling pattern obtained with D10 and D10^Δ*pepc*^-infected RBC with ^13^C-U-D-glucose, ^13^C-bicarbonate and ^13^C,^15^N-U-glutamine has allowed us to construct a scheme of the likely metabolic pathways operating in intermediary metabolism of asexual erythrocytic stages of *P. falciparum* and to postulate how the D10^Δ*pepc*^ mutants have adapted to facilitate survival *in vitro*. We propose (summarised in Fig. 7) that in wild type parasites the OAA resulting from PEPC activity is converted to malate that enters the mitochondrion where it is converted to OAA through the action of MQO. The presence of a cytosolic MDH is well established, a malate/ketoglutarate antiporter has been reported and the presence of MQO is confirmed [28], [42], [43]. The OAA generated probably can leave the mitochondrion, via a transporter postulated previously [39], [43], to complete an abbreviated, parasite-specific, malate shuttle. An alternative possibility postulated recently [6] is that it is citrate that exits the mitochondrion and this is subsequently converted to OAA and acetyl-CoA by citrate synthase II in a citrate lyase-like reaction, but there is no experimental evidence available to date to support this suggestion. Whichever of these happens, the shuttle leads to oxidation of cytosolic NADH, pivotal in maintaining cytosolic redox balance. The importance of this shuttle itself rather than the TCA cycle *per se* is emphasised by the finding that chemical ablation of the TCA cycle using fluoroacetate, which inhibits the aconitase step and thus is subsequent to OAA/citrate formation in the malate shuttle, had no inhibitory effect upon growth of erythrocytic stages of the parasite [3]. An additional factor is that the electrons transferred into the mitochondrion through malate are passed into the ubiquinone pool, which helps maintain mtETC. As the loss of PEPC resulting in no flux into malate from glucose does not affect the mtETC electrochemical gradient (Fig. S4) nor, apparently, the activity of complex III or DHOD (Figs. 4,5C, Table S7), it seems that the reduced contribution of MQO to the mtETC in these erythrocytic stages of D10^Δ*pepc*^ can be compensated for by other inputs. Our findings that there are elevated levels of both DHAP/glyceraldehyde 3-phosphate and glycerol 3-phosphate in the mutants and that they are as strongly labelled with ^13^C in the mutants as in D10, despite a reduced rate of glucose consumption, could mean that there is an increased flux from glycolytic triose phosphates to glycerol 3-phosphate. This is then potentially converted back to triose phosphate via the mitochondrial glycerol 3-phosphate dehydrogenase [44] feeding electrons into mtETC and so increasing flux, thereby compensating for the reduced flux through MQO. This diversion of glycolytic flux would also result in oxidation of NADH (in the conversion of triose phosphate to glycerol 3-phosphate) and so help to compensate for the lack of this occurring in the MDH step. Caution is required, however, in interpreting the data so far, as the elevated levels of trioses in the mutant parasites could reflect lower rates of their use rather than elevated flux to them. Studies in recent years on the mtETC in erythrocytic *P. falciparum* have indicated that it is more complex than had been understood previously [29], [30] and we suggest that the *pepc* KO line that we have generated and characterised could be an important tool with which to dissect this further.

Another result of the lack of malate generation from glucose and the consequent reduction in malate feeding into TCA metabolism would be a reduced flux through isocitrate dehydrogenase (IDH), considered a major source of the generation of NADPH [45]. The higher levels of ribulose 5-P/ribose 5-P in D10^Δ*pepc*^, together with the heavy labelling of the metabolite despite the lower glucose utilisation, could reflect an increased flux through oxidative PPP, which would result in elevated generation of NADPH, compensating for the lower IDH flux. The finding that sedoheptulose 7-P although apparently elevated in abundance is less heavily labelled by ^13^C in D10^Δ*pepc*^ than in D10 itself could be due to the flux from ribulose 5-P to sedoheptulose 7-P (transketolase activity) not being similarly increased in D10^Δ*pepc*^ as is the flux through the oxidative PPP and also that sedoheptulose 7-P is utilised less, neither of which would necessarily impact upon redox homeostasis. Clearly, however, more analyses will be required to fully elucidate the adaptive changes of the mutant parasites.

Thus the modifications to metabolism that we have found in the D10^Δ*pepc*^ mutant have provided a better understanding and new insights into the crucial function of PEPC of *P. falciparum*. The OAA generated by PEPC feeds into pathways central to maintaining cytosolic redox balance (NADH oxidation by MDH) and the mtETC (via MQO), and generating NADPH (via IDH) for antioxidant and biosynthetic reactions. Together these are major contributions to parasite intermediary and redox metabolism, and our findings suggest that interfering with this could have major repercussions for parasite survival *in vivo*, and consequently PEPC inhibitors should be investigated as possible novel antimalarial agents.

## Materials and Methods

### Materials

WR99210 was a kind gift from Jacobus Pharmaceuticals (USA). 5-Fluorocytosine (5-FC) was obtained from The Royal Infirmary Pharmacy, Glasgow. Blasticidin was purchased from Invitrogen (UK). Plasmids pHH1 and pCC4 were kind gifts from Professor A. F. Cowman (The Walter and Eliza Hall Institute, Melbourne, Australia). Human blood was obtained from the Glasgow Blood Transfusion Services. Specific antibodies, used in western blot experiments, were generated in rabbits using recombinant expressed protein (Eurogentec, Belgium). Secondary antibodies were purchased from Promega. Anti-MDH and anti-AAT antibodies were a kind gift from Professor H. Balaram (Jawaharlal Nehru Centre for Advanced Scientific Research, Bangalore, India). DSM190 was a kind gift from Professor M.A. Phillips (Department of Pharmacology, University of Texas Southwestern Medical Center, Dallas, USA). [8-^3^H]-hypoxanthine (specific activity: 10–30 Ci/mmol) was from American Radiolabeled Chemicals. Other chemicals were purchased from Sigma UK unless otherwise stated.

### Generation of *P. falciparum pepc* knockout construct

The plasmid pCC4 [21] contains two homologous regions, to both the 5′ and 3′ end of the target gene, and was used to generate the pCC4-Δ*pepc* knockout construct. The primers used to amplify the 5′fragment (1–498 bp of the *pepc* ORF) and the 3′ fragment (2440–2939 bp of the *pepc* ORF) are shown in Table S8. The sequence of pCC4-Δ*pepc* was verified (Eurofins MWG Operon).

### Parasite culture, transfection, determination of IC_50_ values, parasite growth rate, and metabolite concentrations in spent media

*P. falciparum* D10 (Papua New Guinea) was cultured according to Trager and Jensen [13] in RPMI 1640 (Invitrogen, UK) containing 11 mM glucose, 0.5% (w/v) Albumax II (Invitrogen, UK), 200 µM hypoxanthine, 20 µg/ml gentamycin (PAA, UK) (designated routine medium) in human erythrocytes between 0.5% and 5% haematocrit. Parasite cultures were maintained under an atmosphere of reduced oxygen (1% oxygen, 3% CO_2_ and 96% nitrogen). Parasites were synchronised using sorbitol [46] and freed from erythrocytes using saponin [47]. Parasitaemia was determined using Giemsa-stained thin smears. Transfection of into *P. falciparum* erythrocytic stages was performed as described previously [48]. Transfectants were selected with 2.5 µg/ml blasticidin and they were maintained in either routine medium (which does not contain added malate) or routine medium supplemented with 5 mM malate (malate medium). Before cloning by limiting dilution according to Kirkman et al. [49], pCC4-Δ*pepc* transfectants were subjected to negative selection with 1 µM 5-FC.

Growth rates of D10 and D10^Δ*pepc*^ were determined as described by Günther et al. [50]. For the rescue experiments, D10^Δ*pepc*^ were cultured in routine medium for 9 days, synchronised, diluted to 1% parasitaemia and cultured for 5 days in routine medium or routine medium supplemented with increasing concentrations of malate (0.5 mM to 5 mM), 5 mM aspartate, 5 mM fumarate, 5 mM succinate, 5 mM glycerol, 5 mM glutamine or 0.5 mM citrate. D10 were grown in the same media as controls. The cultures were diluted 1∶5 on day 3 and the parasitaemia of 1000 RBC was determined. The effects of L-cycloserine, DSM190 and atovaquone on the viability of the parasites were determined by measuring the incorporation of [^3^H]-hypoxanthine in the presence of increasing drug concentrations according to Desjardins et al. [51]. D10 and D10^Δ*pepc*^ were maintained in either routine (D10^Δ*pepc*^ for 9 days) or malate medium and synchronised two days prior to the experiment. IC_50_ values were calculated by nonlinear regression of the sigmoidal dose-response equation using GraFit version 7 (Erithacus, UK).

The concentrations of glucose, lacatate, glutamine, succinate, malate and fumarate in the spent media from RBC, D10 and D10^Δ*pepc*^ cultures were assayed using the following kits: glucose (Megazyme D-Glucose-HK), lactate (Megazyme D-/L-Lactic Acid (D-/L-Lactate) (Rapid)), glutamine (Megazyme L-Glutamine/Ammonia (Rapid)), malate (Megazyme L-Malic Acid), succinate (Megazyme Succinic Acid) and fumarate (Sigma Fumarate Assay Kit). All assays were carried out as per manufacturer's recommendation.

### Targeted metabolomics using ^13^C-metabolites

Targeted metabolomics was performed with D10 and D10^Δ*pepc*^ cultured in routine medium for 9 days. After synchronisation, the RBC number of D10 and D10^Δ*pepc*^ infected cultures was determined using a Scepter 2.0 Handheld Automated Cell Counter (Millipore, UK) and adjusted to 6% parasitaemia and 1% haematocrit with culture medium containing ^13^C-labelled metabolites. Uninfected RBC of the same batch were used as control at the same haematocrit. Either glucose, bicarbonate or glutamine were replaced with 11 mM ^13^C-U- D-glucose (99%, CK Gas Products Ltd., UK), 0.2% ^13^C sodium bicarbonate (99%, CK Gas Products, UK) or 2 mM ^13^C, ^15^N-U-glutamine (99%, CK Gas Products, UK), respectively, and cultures were incubated for 28 h post-synchronisation. Spent medium was collected and metabolites were extracted in HPLC-grade chloroform∶methanol (1∶3 v/v). Metabolism of parasitized RBC was quenched to 4°C using a dry ice/70% ethanol bath [52]. Infected RBC were washed in ice-cold PBS and 3.5×10^8^ cells were used for metabolite extraction using HPLC-grade chloroform∶methanol∶water (1∶3∶1 v/v/v). Samples were sonicated using a sonicating waterbath and then metabolites extracted for 1 h at 4°C under shaking conditions. The extracted samples were centrifuged at 16,000 g for 15 min at 4°C and the supernatants were transferred into glass vials and stored at −80°C until LC-MS analysis. Late stage D10 cultures obtained 28 hours post-sorbitol synchronisation were also enriched by magnetic purification using the Vario MACS magnetic system and LS columns (Miltenyi Biotec) [31], [53], [54]. This procedure resulted in an accumulation of infected RBC to 90% and metabolites of 3×10^7^ infected RBC were analysed.

### Liquid chromatography-mass spectrometry analyses

All samples were analysed using a liquid chromatography (LC) mass spectrometry system (MS) (Ultimate 3000 LC system, Dionex, UK, connected to an Exactive mass spectrometer, Thermo Scientific, Germany). The LC system was controlled using Chromeleon (Dionex, UK), and the MS was controlled by the software Xcalibur (Thermo Scientific, Germany), which allowed recording data for both positive and negative ionisation mode. Separation of analytes was achieved via a ZIC-pHILIC chromatography column (150 mm×4.6 mm×5 µm; Sequant, Uemå, Sweden) by a two solvent system consisting of solvent A: 20 mM ammonium carbonate and solvent B: acetonitrile. The chromatography conditions are summarised in Table S9. Both positive and negative mode spectra were acquired using 3 microscans over a scan range of 75.0–1200.0 m/z at a resolution of 50,000 (FWHM at 500 m/z) with an automatic gain control (AGC) target of 1×10^6^ and a maximum inject time of 250 milliseconds. Specific instrument settings are supplied in the supporting information.

### Data analysis

Data processing initially involved centroiding and converting vendor-specific raw LC-MS files into the mzXML open format. Chromatographic peaks in these files were extracted using the detection algorithm from XCMS [55] and stored in corresponding PeakML files [56]. Subsequently, PeakML files representing replicates were aligned and combined using mzMatch.R [57] after filtering out all peaks that were not reproducibly detected. The combined PeakML files were subjected to additional noise filtering, gap-filling and metabolite identification steps, using authentic metabolic standards for all metabolites of interest to ensure reliable identification. The proportion of each metabolite labelled with the stable isotopes was determined (see Figure S6) together with information on the overall abundance of metabolites present in the respective parasite extract samples or the culture medium at the time of harvesting of the parasites. The PeakML file obtained after filtering and identification was scanned for labelled metabolites using the PeakML.Isotope.TargettedIsotopes function of mzMatch-ISO [58]. Further details are provided in the supporting information.

### Genomic DNA and Southern blotting

Genomic DNA was isolated from saponin isolated parasites using the QIAamp DNA Mini Kit (Qiagen, UK). 1–3 µg of genomic DNA, digested with SpeI, were separated on a 0.8% agarose gel and blotted onto positively charged nylon membrane (GE Healthcare, UK). The blots were pre-hybridized and probed at 55°C. Probes were labelled with thermostable alkaline phosphatase using the AlkPhos Direct Labeling and Detection System (VWR, UK) and successive washes were performed according to the manufacturer's instructions at 55°C. The blots were exposed to hypersensitive autoradiography film (VWR, UK) for 1–16 h.

### Homology modelling of *P. falciparum* PEPC

Homology modelling of *P. falciparum* PEPC (*Pf*PEPC) was performed using the I-TASSER server for protein structure and function prediction (http://zhanglab.ccmb.med.umich.edu/I-TASSER/) [59], [60]. The top three templates used for structure prediction of *Pf*PEPC were the PEPC from *E. coli* (*Ec*PEPC) (PDB entry 1JQN, [61]), PEPC from maize (*Zm*PEPC) (PDB entry 1JQO, [61]) and the archaeal-type PEPC (PDB entry 3ODM, [62]). Root mean square deviation (RMSD) values showed *Pf*PEPC to be most similar to *Ec*PEPC (0.56 Å), followed by *Zm*PEPC (2.49 Å) and archaeal-PEPC (3.80 Å). Based on these templates, five I-TASSER models were obtained for *Pf*PEPC with C-scores ranging from −0.86 to −1.23. The structural alignment of the best model, that is, the model with the highest C-score (−0.86), with the top structural analogue, that is *Ec*PEPC (0.56 Å), was generated using Magic Fit in Swiss-PDB Viewer 4.1.0 (http://www.expasy.org/spdbv/) [63]. This is shown in Fig. 3.

### Statistical analyses

See supporting information for details.

## Supporting Information

Figure S1 **Gene disruption and 3′ replacement of** ***pepc*** **by the pHH1 plasmid.** (A) Schematic diagram of the endogenous *pepc* gene locus in D10 wild type parasites, the pHH1-Δ*pepc* plasmid and the recombined *pepc* locus following single cross over recombination between the plasmid and an 1113 bp region of *pepc* (D10^Δ*pepc*^). The plasmid contains a human *dihydrofolate reductase* (*hDHFR*) selectable marker under control of the *P. falciparum calmodulin* promoter (*cam* 5′) and flanked by the *P. falciparum histidine rich protein 2* terminator (*hrp2* 3′), a region homologous to *pepc* (Δ*pepc*), an artificial 3′ UTR (*P. berghei dihydrofolate reductase/thymidylate synthase* 3′UTR, *PbDT* 3′) and an artificial stop codon (Stop*). AccI restriction sites and sizes of the resulting diagnostic DNA fragments are indicated (in bold). (B) Similar scheme for the integration of the pHH1-3′*pepc* plasmid, resulting in D10^3′*pepc*^, which includes a functional *pepc* gene. Diagnostic NspI sites and resulting fragment sizes are indicated. (C) Two independent Southern blots of D10 and D10^Δ*pepc*^, probed with the Δ*pepc* DNA fragment. c2 and c3 refer to the WR selection cycles, in which the parasites are grown without drug for 3 weeks and then subjected to WR selection. Plasmid (6.9 kb) is absent and seemed to be integrated in cycle 2 and 3 (D10^Δ*pepc*^ c2 and D10^Δ*pepc*^ c3). However, only the 2.9 kb integration fragment is present. The 5.7 kb fragment is not detected, but instead a fragment of ∼4 kb is present. Endogenous *pepc* (1.8 kb) is present in all D10^Δ*pepc*^ lines and D10. (D) Southern blot of pHH1-3′*pepc*, D10 and D10^3′*pepc*^, probed with the 3′*pepc* DNA fragment. Integration fragments (2.3 and 4.4 kb) and the fragment corresponding to pHH1-3′*pepc* (1.2 kb) are detected in D10^3′*pepc*^ c1. The fragment indicative of endogenous *pepc* (5.5 kb) has disappeared, but is visible in D10. (E) Pulse field gel electrophoresis of D10, D10^Δ*pepc*^ and D10^3′*pepc*^. In the left panel, the ethidium bromide-stained gel, indicating chromosomes 11 to 14, is shown. In the right panel, the southern blot is shown, probed with either the Δ*pepc* DNA fragment or the *hDHFR* DNA fragment. The Δ*pepc* probe detects integration of the pHH1- Δ*pepc* plasmid or endogenous *pepc*, which is located on chromosome 14 and present in all lines. A signal is also present in the band representing chromosomes 1–10, indicative of random integration of pHH1- Δ*pepc* (D10^Δ*pepc*^ c2 and D10^Δ*pepc*^ c3). This is confirmed with the *hDHFR* probe, which also detects integration of the pHH1- Δ*pepc* plasmid in the smaller chromosomes. The pHH1-3′*pepc* plasmid did integrate in the correct gene locus on chromosome 14 (D10^3′*pepc*^ c2).
(TIF)

Figure S2 **ClustalW alignment (1.83) multiple sequence alignment.** CLUSTAL W (1.83) multiple sequence alignment. (*), identical residues; (:), strong conservation between replaced amino acids; (.), weak conservation between replaced amino acids; (-), gaps to maximise alignment; bold red letters: active site residues; bold blue letters: residues involved in binding of hexose 6-phosphate; bold green letters: determinants that distinguish C4 or C3 plant PEPCs; N-terminal blue blocked: site of phosphorylation of plant PEPCs – the serine residue is absent from *P. falciparum* PEPC and *E. coli* PEPC; C-terminal blue blocked: unique, parasite-specific serine residue found to be phosphorylated in phosphoproteome of *P. falciparum*; green blocked: Loop I and II both involved in catalytic activity; yellow blocked: C-terminal catalytic peptide. At, *Arabidopsis thaliana* PEPC 3 (NP_188112); Ec, *Escherichia coli* PEPC (B7MR83); Fp, *Flaveria pringlei* PEPC (Q01647); Ft, *F. trinervia* (P30694); Pf, *P. falciparum* PEPC (PF3D7_1426700); Zm, *Zea mays* PEPC (ACJ38542).
(DOCX)

Figure S3 **Western blot analyses of expression of MDH and AAT.** The left panel (A) shows representative western blots of 10 µg parasite extract of D10 and two clones of D10^Δ*pepc*^ (D10^Δ*pepc*^-1 and D10^Δ*pepc*^-2), either in malate or routine medium, with antibodies against (A) MDH (34 kDa) or (B) AAT (42 kDa). D10^Δ*pepc*^- 1 and D10^Δ*pepc*^-2 routine were cultured in routine medium for 9 days prior to the extraction. The loading control is an antibody against 2-Cys peroxiredoxin (2-CP, 22 kDa) and is shown underneath each blot. The right panel (B) shows the respective graphs (mean ± S.E.M.) of the densitometry analyses of 3 or 4 independent extracts. Relative protein expression was calculated by comparing the intensity of the MDH or AAT bands with the 2-CP loading control, normalised to D10.
(TIF)

Figure S4 **Mitochondrial membrane potential of D10 and D10^Δ^** ^***pepc***^ **.** The mitochondrial membrane potential was assessed by the accumulation of MitoTracker Red CMXRos (red fluorescence) and is shown in the right panel together with a nuclear stain (Hoechst 33258, blue fluorescence). DIC images of the live cells are shown in the left panel. D10^Δ*pepc*^ was cultured in routine medium for 9 days and as control for a collapsed mitochondrial membrane potential, D10 was pre-treated with 500 nM valinomycin (bottom panel).
(TIF)

Figure S5 **Labelling of D10 and D10^Δ^** ^***pepc***^ **with ^13^C,^15^N-U-glutamine.** (A) Schematic representation of glutamine utilisation in *P. falciparum* based on the utilisation of ^13^C,^15^N-U-glutamine and distribution of ^13^C carbons into some major metabolic intermediates of the TCA cycle. (B) The parasites use glutamine to generate α-ketoglutarate (^13^C-5-labelled), which is translocated into the mitochondrion, where it is converted to succinate (^13^C-4-labelled). The flux into fumarate and malate is apparently low and only small amounts of fumarate (^13^C-4-labelled) and malate (^13^C-4-labelled) are detectable.
(TIF)

Figure S6 **Analysis of metabolite labelling with ^13^C-isotopes.** The schematic shows the abundance of different species of a hypothetical metabolite containing 6-carbons after extraction from a biological sample. Open circles display unlabelled (^12^C) carbon, red-filled circles represent ^13^C- carbon. Peak shapes and area were assessed and retention times confirmed manually before relative incorporation of heavy isotopes into the metabolite was calculated from the peak areas of each labelled metabolite species as shown in A. Natural abundance of ^13^C-1species is represented by the bar in the 1^13^C column in A and the relative proportions of the metabolite containing different numbers of ^13^C atoms was calculated and displayed as shown in B.
(TIF)

Table S1 **Peak areas of selected metabolites after labelling infected RBC (6% parasitaemia) with ^13^C-U-L-glucose.** Parasitaemia was 6%, labelling was performed for 28 h before cells were harvested and metabolites extracted. Shown are the peak areas calculated from the negative ionisation mode obtained by LC-MS. RBC, D10 wild type and D10^Δ*pepc*^ as well as spent media of all three cell types were analysed in this experiment. The data represent three biological replicates.
(XLS)

Table S2 **Peak areas of selected metabolites after labelling infected RBC (90% parasitaemia) with ^13^C-U-L-glucose.** Parasitaemia was 90%, labelling was performed for 28 h before RBC and D10-parasitised RBC were harvested and metabolites extracted. Shown are the peak areas calculated from the negative ionisation mode obtained by LC-MS. The data represent three biological replicates.
(XLS)

Table S3 **Peak areas of selected metabolites after labelling infected RBC (6% parasitaemia) with ^13^C-bicarbonate.** Parasitaemia was 6%, labelling was performed for 28 h before cells were harvested and metabolites extracted. Shown are the peak areas calculated from the negative ionisation mode obtained by LC-MS. RBC, D10 wild type and D10^Δ*pepc*^ as well as spent media of all three cell types were analysed in this experiment. The data represent three biological replicates.
(XLS)

Table S4 **Peak areas of selected metabolites after labelling infected RBC (90% parasitaemia) with ^13^C-bicarbonate.** Parasitaemia was 90%, labelling was performed for 28 h before RBC and D10-parasitised RBC were harvested and metabolites extracted. Shown are the peak areas calculated from the negative ionisation mode obtained by LC-MS. The data represent three biological replicates.
(XLS)

Table S5 **Peak areas of selected metabolites after labelling infected RBC (6% parasitaemia) with ^13^C, ^15^N-U-glutamine.** Parasitaemia was 6%, labelling was performed for 28 h before cells were harvested and metabolites extracted. Shown are the peak areas calculated from the negative ionisation mode obtained by LC-MS. RBC, D10 wild type and D10^Δ*pepc*^ as well as spent media of all three cell types were analysed in this experiment. The data represent three biological replicates.
(XLSX)

Table S6 **Peak areas of selected metabolites after labelling infected RBC (90% parasitaemia) with ^13^C, ^15^N-U-glutamine.** Parasitaemia was 90%, labelling was performed for 28 h before RBC and D10-parasitised RBC were harvested and metabolites extracted. Shown are the peak areas calculated from the negative ionisation mode obtained by LC-MS. The data represent three biological replicates.
(XLSX)

Table S7 **Peak areas of pyrimidine biosynthesis intermediates and purines after labelling infected RBC (6% parasitaemia) with ^13^C-bicarbonate or ^13^C, ^15^N-U-glutamine.** Parasitaemia was 6%, labelling was performed for 28 h before cells were harvested and metabolites extracted. Shown are the peak areas calculated from the negative ionisation mode obtained by LC-MS. In both experiments, labelled and unlabelled datasets were generated and analysed by LC-MS. The data represent D10 (D10 unlabelled), D10-Lab (D10 labelled with heavy isotope metabolites), PEPCKO (D10^Δ*pepc*^ (unlabelled), and PEPCKO-Lab (D10^Δ*pepc*^ labelled with heavy isotope metabolites). The data represent three biological replicates.
(XLSX)

Table S8 **Primers used in this study.** Restriction sites are shown in bold letters.
(DOCX)

Table S9 **Chromatography conditions LC-MS.** The detailed profile of the solvents used for the chromatography elution.
(DOCX)

Text S1 **Supporting materials and methods and references.**
(DOCX)
