# Supplementary figures and images for: Phosphoenolpyruvate Carboxylase Identified as a Key Enzyme in Erythrocytic Plasmodium falciparum Carbon Metabolism
Source: PLoS Pathog. 2014 Jan 16;10(1):e1003876. doi: 10.1371/journal.ppat.1003876 (PMC3894211; doi:10.1371/journal.ppat.1003876)

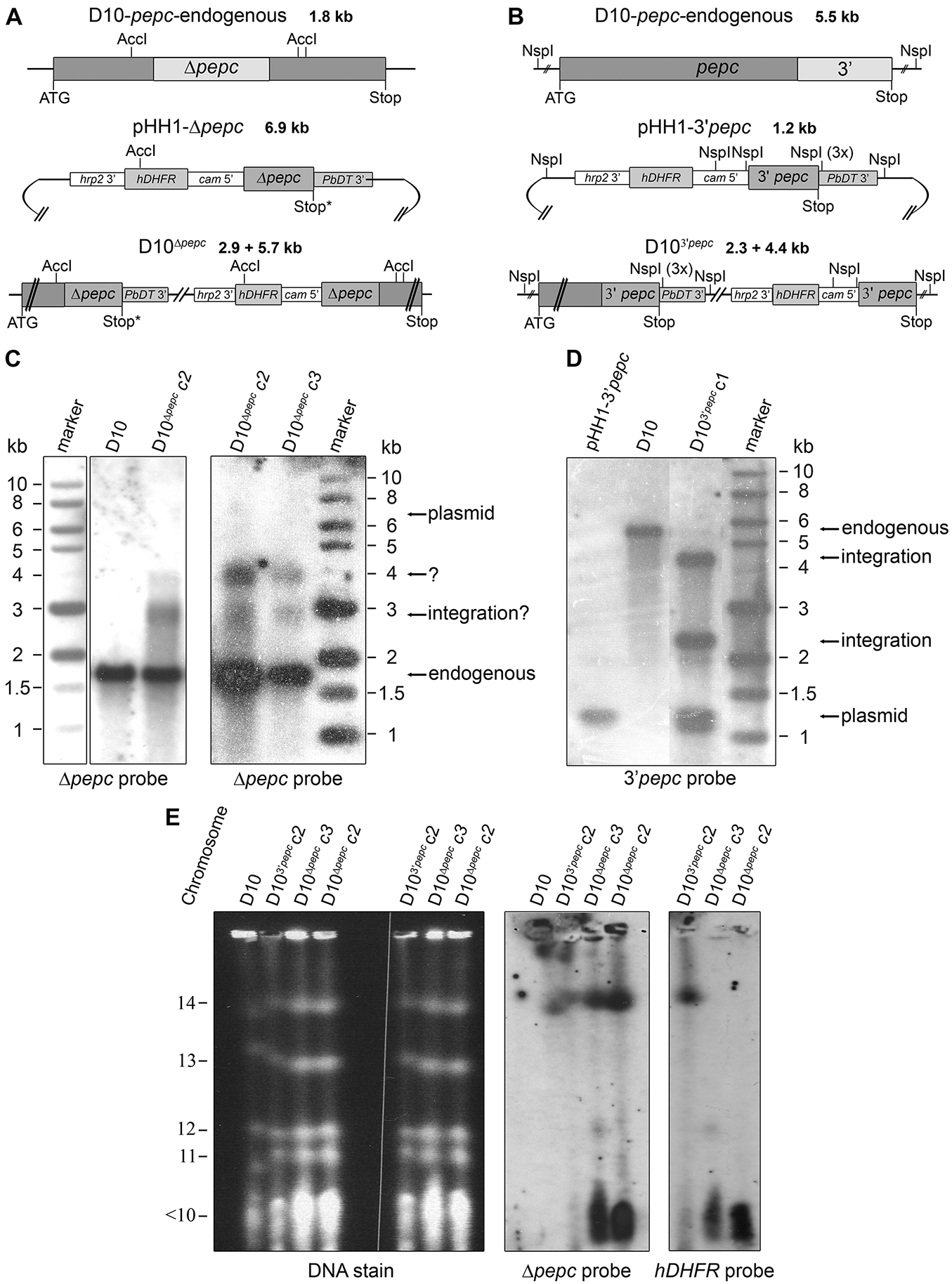

Supplement: Figure S1 — Gene disruption and 3′ replacement of pepc by the pHH1 plasmid. (A) Schematic diagram of the endogenous pepc gene locus in D10 wild type parasites, the pHH1-Δpepc plasmid and the recombined pepc locus following single cross over recombination between the plasmid and an 1113 bp region of pepc (D10Δpepc). The plasmid contains a human dihydrofolate reductase (hDHFR) selectable marker under control of the P. falciparum calmodulin promoter (cam 5′) and flanked by the P. falciparum histidine rich protein 2 terminator (hrp2 3′), a region homologous to pepc (Δpepc), an artificial 3′ UTR (P. berghei dihydrofolate reductase/thymidylate synthase 3′UTR, PbDT 3′) and an artificial stop codon (Stop*). AccI restriction sites and sizes of the resulting diagnostic DNA fragments are indicated (in bold). (B) Similar scheme for the integration of the pHH1-3′pepc plasmid, resulting in D103′pepc, which includes a functional pepc gene. Diagnostic NspI sites and resulting fragment sizes are indicated. (C) Two independent Southern blots of D10 and D10Δpepc, probed with the Δpepc DNA fragment. c2 and c3 refer to the WR selection cycles, in which the parasites are grown without drug for 3 weeks and then subjected to WR selection. Plasmid (6.9 kb) is absent and seemed to be integrated in cycle 2 and 3 (D10Δpepc c2 and D10Δpepc c3). However, only the 2.9 kb integration fragment is present. The 5.7 kb fragment is not detected, but instead a fragment of ∼4 kb is present. Endogenous pepc (1.8 kb) is present in all D10Δpepc lines and D10. (D) Southern blot of pHH1-3′pepc, D10 and D103′pepc, probed with the 3′pepc DNA fragment. Integration fragments (2.3 and 4.4 kb) and the fragment corresponding to pHH1-3′pepc (1.2 kb) are detected in D103′pepc c1. The fragment indicative of endogenous pepc (5.5 kb) has disappeared, but is visible in D10. (E) Pulse field gel electrophoresis of D10, D10Δpepc and D103′pepc. In the left panel, the ethidium bromide-stained gel, indicating chromosomes 11 to 14, is shown [file ppat.1003876.s001.tif]

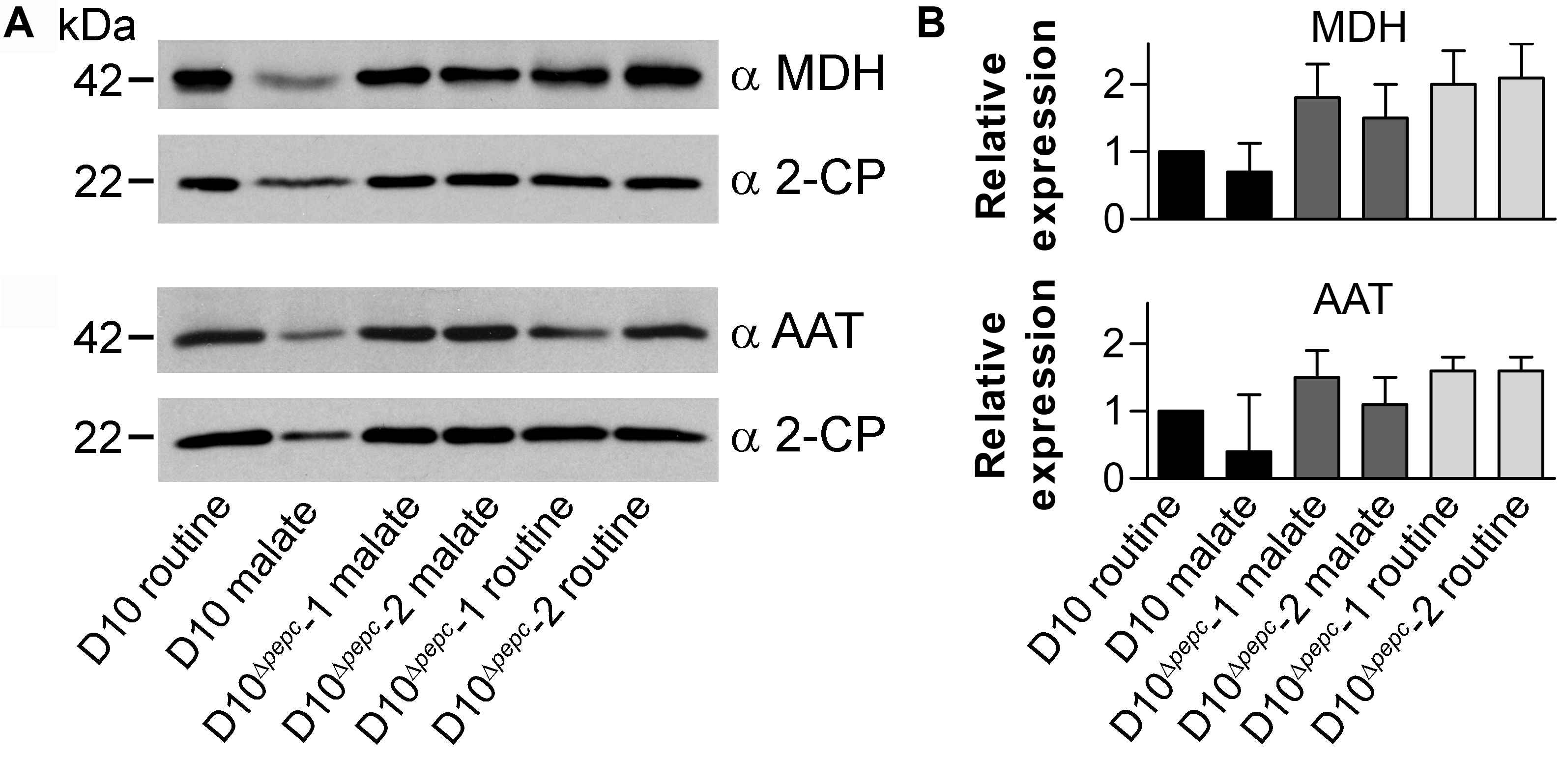

Supplement: Figure S3 — Western blot analyses of expression of MDH and AAT. The left panel (A) shows representative western blots of 10 µg parasite extract of D10 and two clones of D10Δpepc (D10Δpepc-1 and D10Δpepc-2), either in malate or routine medium, with antibodies against (A) MDH (34 kDa) or (B) AAT (42 kDa). D10Δpepc- 1 and D10Δpepc-2 routine were cultured in routine medium for 9 days prior to the extraction. The loading control is an antibody against 2-Cys peroxiredoxin (2-CP, 22 kDa) and is shown underneath each blot. The right panel (B) shows the respective graphs (mean ± S.E.M.) of the densitometry analyses of 3 or 4 independent extracts. Relative protein expression was calculated by comparing the intensity of the MDH or AAT bands with the 2-CP loading control, normalised to D10. (TIF) [file ppat.1003876.s003.tif]

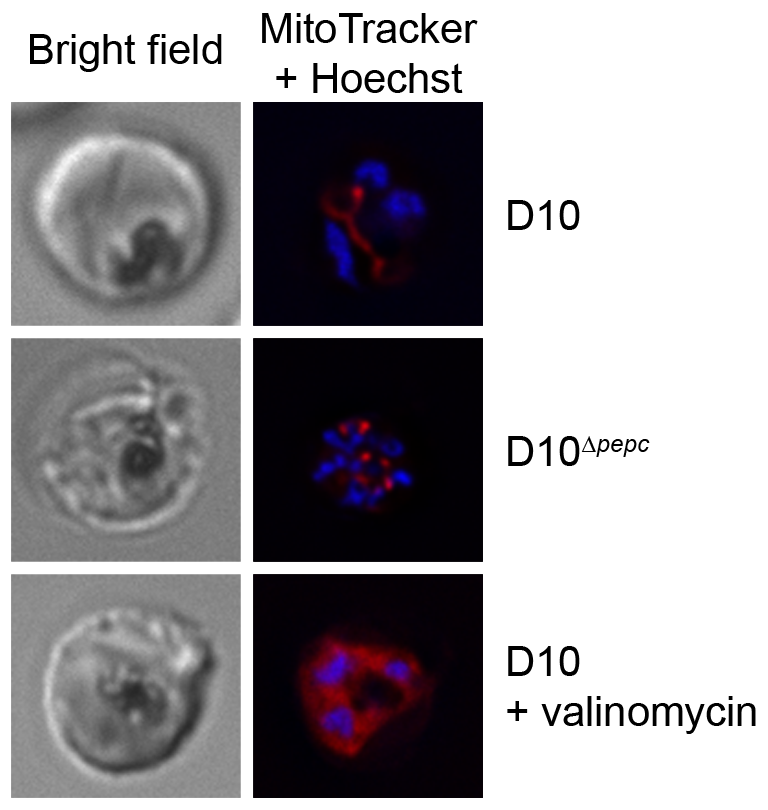

Supplement: Figure S4 — Mitochondrial membrane potential of D10 and D10Δ pepc . The mitochondrial membrane potential was assessed by the accumulation of MitoTracker Red CMXRos (red fluorescence) and is shown in the right panel together with a nuclear stain (Hoechst 33258, blue fluorescence). DIC images of the live cells are shown in the left panel. D10Δpepc was cultured in routine medium for 9 days and as control for a collapsed mitochondrial membrane potential, D10 was pre-treated with 500 nM valinomycin (bottom panel). (TIF) [file ppat.1003876.s004.tif]

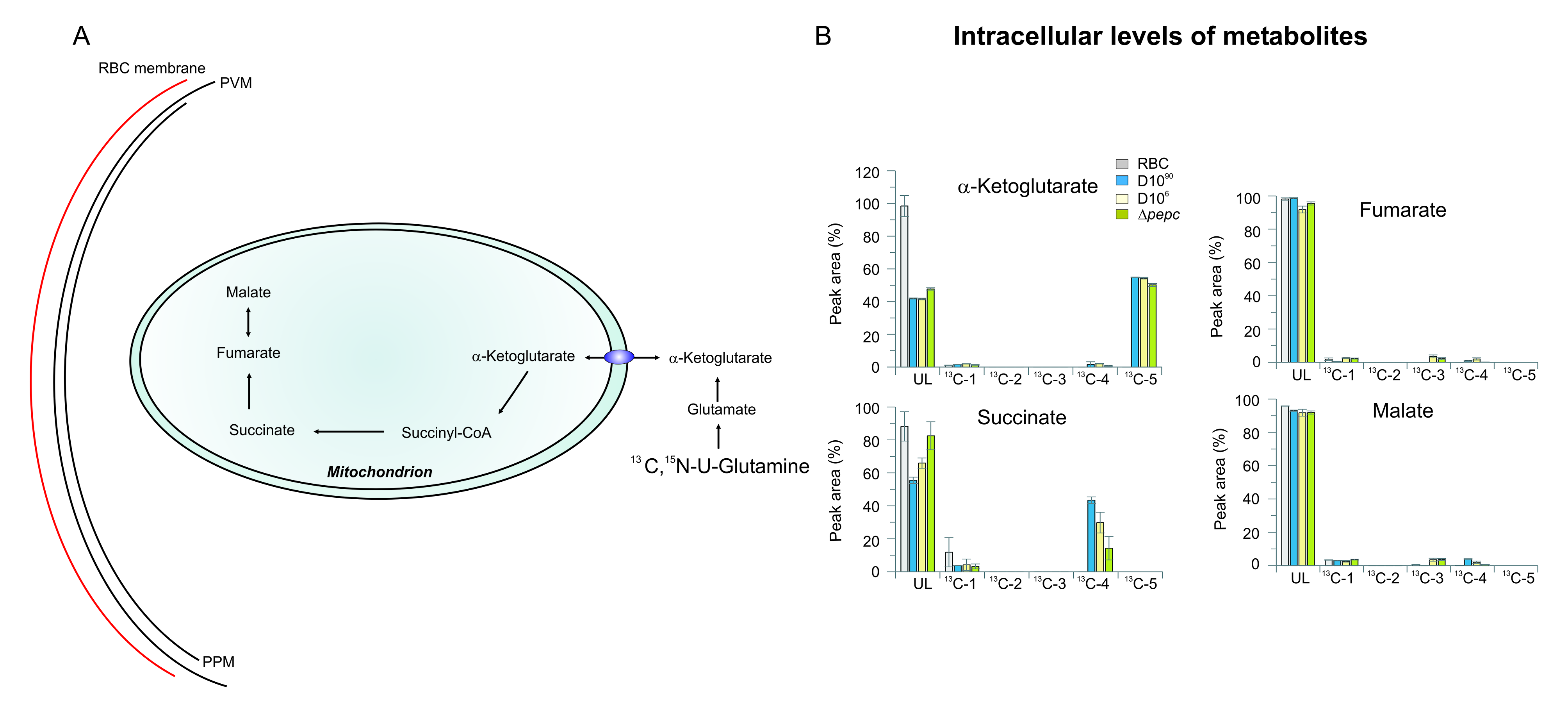

Supplement: Figure S5 — Labelling of D10 and D10Δ pepc with 13C,15N-U-glutamine. (A) Schematic representation of glutamine utilisation in P. falciparum based on the utilisation of 13C,15N-U-glutamine and distribution of 13C carbons into some major metabolic intermediates of the TCA cycle. (B) The parasites use glutamine to generate α-ketoglutarate (13C-5-labelled), which is translocated into the mitochondrion, where it is converted to succinate (13C-4-labelled). The flux into fumarate and malate is apparently low and only small amounts of fumarate (13C-4-labelled) and malate (13C-4-labelled) are detectable. (TIF) [file ppat.1003876.s005.tif]

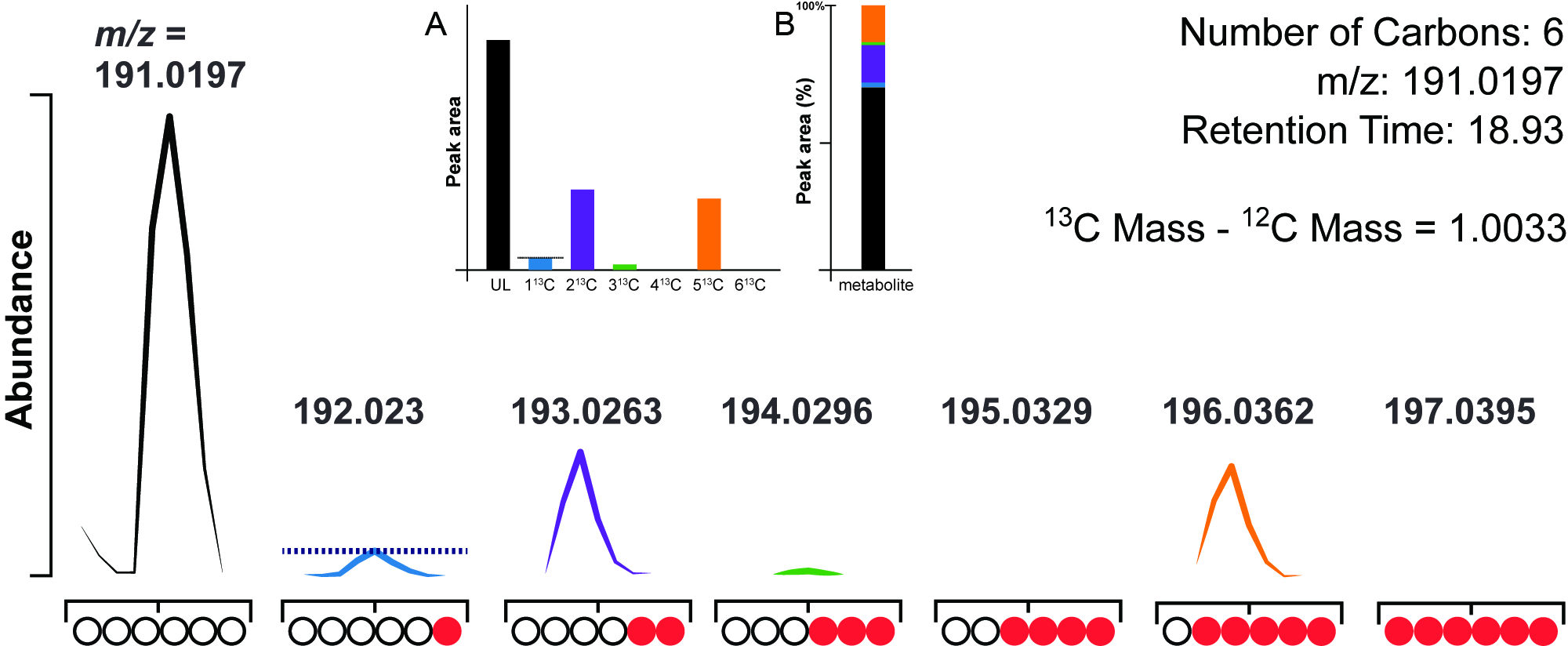

Supplement: Figure S6 — Analysis of metabolite labelling with 13C-isotopes. The schematic shows the abundance of different species of a hypothetical metabolite containing 6-carbons after extraction from a biological sample. Open circles display unlabelled (12C) carbon, red-filled circles represent 13C- carbon. Peak shapes and area were assessed and retention times confirmed manually before relative incorporation of heavy isotopes into the metabolite was calculated from the peak areas of each labelled metabolite species as shown in A. Natural abundance of 13C-1species is represented by the bar in the 113C column in A and the relative proportions of the metabolite containing different numbers of 13C atoms was calculated and displayed as shown in B. (TIF) [file ppat.1003876.s006.tif]
