# Supplementary material for: Phosphoenolpyruvate Carboxylase Identified as a Key Enzyme in Erythrocytic Plasmodium falciparum Carbon Metabolism
Source: PLoS Pathog. 2014 Jan 16;10(1):e1003876. doi: 10.1371/journal.ppat.1003876 (PMC3894211; doi:10.1371/journal.ppat.1003876)
Supplement: Figure S2 — ClustalW alignment (1.83) multiple sequence alignment. CLUSTAL W (1.83) multiple sequence alignment. (*), identical residues; (:), strong conservation between replaced amino acids; (.), weak conservation between replaced amino acids; (-), gaps to maximise alignment; bold red letters: active site residues; bold blue letters: residues involved in binding of hexose 6-phosphate; bold green letters: determinants that distinguish C4 or C3 plant PEPCs; N-terminal blue blocked: site of phosphorylation of plant PEPCs – the serine residue is absent from P. falciparum PEPC and E. coli PEPC; C-terminal blue blocked: unique, parasite-specific serine residue found to be phosphorylated in phosphoproteome of P. falciparum; green blocked: Loop I and II both involved in catalytic activity; yellow blocked: C-terminal catalytic peptide. At, Arabidopsis thaliana PEPC 3 (NP_188112); Ec, Escherichia coli PEPC (B7MR83); Fp, Flaveria pringlei PEPC (Q01647); Ft, F. trinervia (P30694); Pf, P. falciparum PEPC (PF3D7_1426700); Zm, Zea mays PEPC (ACJ38542). (DOCX) [file ppat.1003876.s002.docx]

Pf MSNNHNNFIYCSGTEDDHVNNAETGGKEDEKLKFLIEGKMIEKKGCVDFIKPLKDDIKALDFLLFDMLKDNLPNNLFEILCTIHDLSETYSENPNDDNFLSLKNCIYNLKDEYLGTIVNA 120

Zm MASTKAP------------GPGEKHH**S**IDAQLRQLVPGKVSEDDKLIEYDAL------LVDRF-LNILQDLHGPSLREFVQECYEVSADYEGKGDTTKLGELGAKLTGLAPADAILVASS 101

Ft MANRN----------------VEKLA**S**IDAQLRLLVPGKVSEDDKLVEYDAL------LLDKF-LDILQDLHGEDLKEAVQQCYELSAEYEGKHDPKKLEELGSLLTSLDTGDSIVIAKA 97

Fp MANRN----------------LEKLA**S**IDAQLRLLVPGKVSEDDKLIEYDAL------LLDKF-LDILQDLHGEDLKEAVQECYELSAEYEGKHDPKKLEELGSVLTSLDPGDSIVIAKA 97

At MAGRN----------------IEKMA**S**IDAQLRQLVPAKVSEDDKLVEYDAL------LLDRF-LDILQDLHGEDLRETVQELYELSAEYEGKREPSKLEELGSVLTSLDPGDSIVISKA 97

Ec MNEQ---------------------------------------YSALRSNVS------MLGKVLGETIKDALGEHILERVETIRKLSKSSRAGNDAN-RQELLTTLQNLSNDELLPVARA 74

* : :. . : ::* : * : .:* : .* : .* : :

Pf FGHMCVISNFAEWAHRGRRRKA-FDKSFIPNDKIYGSVNETLKGTFNILIQN-GFQLNDIYEQLCNQTIEFVLTT**H**PTQAI**RT**SLLKNYIRLGELLLKLDNTDKELYKKKLLYDNLKTNL 238

Zm ILHMLNLANLAEEVQIAHRRRNSKLKKGGFADEGSATTESDIEETLKRLVSEVGKSPEEVFEALKNQTVDLVFTA**H**PTQSA**RR**SLLQKNARIRNCLTQLNAKDITDDDKQELDEALQREI 221

Ft FSHMLNLANLAEELQIAYRRRI-KLKSGDFADEANATTESDIEETFKRLVHKLNKSPEEVFDALKNQTVELVLTA**H**PTQSV**RR**SLLQKHGRIRNCLAQLYAKDITPDDKQELDEALHREI 216

Fp FSHMLNLANLAEEVQIAYRRRI-KLKRGDFADEANATTESDIEETFKKLVLKLNKSPEEVFDALKNQTVDLVLTA**H**PTQSV**RR**SLLQKHGRIRNCLAQLYAKDITPDDKQELDEALHREI 216

At FSHMLNLANLAEEVQIAHRRRIKKLKKGDFVDESSATTESDIEETFKRLVSDLGKSPEEIFDALKNQTVDLVLTA**H**PTQSV**RR**SLLQKHGRIRDCLAQLYAKDITPDDKQELDESLQREI 217

Ec FSQFLNLANTAEQYHSISPK-----------GE-AASNPEVIARTLRKLKNQPELSEDTIKKAVESLSLELVLTA**H**PTEIT**RR**TLIHKMVEVNACLKQLDNKDIADYEHNQLMRRLRQLI 182

: :: ::* ** : : .: .: . : *:. * . . : : . : . ::::*:*:***: * :*::: .: * :* .* .:: * *: :

Pf LSSWKTDVIR**R**IKPTPIDEAISLVDIVENCIFYRIPNIIRYIDNVLFEYNLP-PVKLNSKVCIFSSWAGGDRDG-NPFVLPETTKYVCYMNKIRGCELFIPMIEILIRDLTLHHCTQHFR 356

Zm QAAFRTDEIR**R**AQPTPQDEMRYGMSYIHETVWKGVPKFLRRVDTALKNIGINERLPYNVSLIRFSSWMGGDRDG-NPRVTPEVTRDVCLLARMMAANLYIDQIEELMFELSMWRCNDELR 340

Ft QAAFRTDEIR**R**TPPTPQDEMRAGMSYFHETIWKGVPKFLRRVDTALKNIGINERFPYNAPLIQFSSWMGGDRDG-NPRVTPEVTRDVCLLARMMTSNMYFSQIEDLMIEMSMWRCNSELR 335

Fp QAAFRTDEIR**R**TPPTPQDEMRAGMSYFHETIWKGVPKFLRRVDTALKNIGINERVPYNAPLIQFSSWMGGDRDGKHPRVTPEVTRDVCLLARMMASNMYFSQIEDLMFEMSMWRCNSELR 336

At QAAFRTDEIR**R**TPPTPQDEMRAGMSYFHETIWKGVPKFLRRVDTALKNIGIDERVPYNAPLIQFSSWMGGDRDG-NPRVTPEVTRDVCLLARMMAANLYYNQIENLMFELSMWRCTDEFR 336

Ec AQSWHTDEIR**K**LRPSPVDEAKWGFAVVENSLWQGVPNYLRELNEQLEE-NLGYKLPVEFVPVRFTSWMGGDRDG-NPNVTADITRHVLLLSRWKATDLFLKDIQVLVSELSMVEATPELL 300

:::** **: *:* ** . ..: :: :*: :* :: * : .: . : *:** ****** :* * .: *: * : : ::: *: *: :::: ... .:

Pf SYVKLLEDEVSEYIFD**K**DHKYLAKKFQWFSPFSKSNKKEIY**R**RALLIVWAKLKSVVEVYKSLISNQRVDEDFKKLMFHNSDEFEEILLECYKSLVESGNTLIAEGYLKDVIRNVKIFGLH 476

Zm VRAEELHSSSGS----**K**VTKYYIE------LWKQIPPNEPY**R**VILGHVRDKLYNTRERARHLLASG-VSEISAESSFTSIEEFLEPLELCYKSLCDCGDKAIADGSLLDLLRQVFTFGLS 449

Ft VRAEELYRTAR-----**K**DVKHYIE------FWKRIPPNQPY**R**VILGDVRDKLYNTRERSRHLLVDG-KSDIPDEAVYTNVEQLLEPLELCYRSLCDCGDHVIADGSLLDFLRQVSTFGLS 443

Fp VRAEELYRTAR-----**R**DVKHYIE------FWKQVPPTEPY**R**VILGDVRDKLYNTRERSRHLLAHG-ISDIPEEAVYTNVEQFLEPLELCYRSLCDCGDRVIADGSLLDFLRQVSTFGLS 444

At VRADELHRNSRK----**D**AAKHYIE------FWKTIPPTEPY**R**VILGDVRDKLYHTRERSRQLLSNG-ISDIPEEATFTNVEQFLEPLELCYRSLCSCGDSPIADGSLLDFLRQVSTFGLS 445

Ec ALVGEE-----------------------------GAAEPY**R**YLMKNLRSRLMATQAWLEARLK-G-EELPKPEGLLTQNEELWEPLYACYQSLQACGMGIIANGDLLDTLRRVKCFGVP 389

. : ** : : :* . . : . : . ::: * * **:** .* **:* * * :*.* **:

Pf LMKLDIRQESEKHISTMNYICQKLNMK-KYSLLNEEQKINFLTDILNSNRPLIPKNIEEEEDVPNDFINVIKTFDVCSQIEDSALGAYIVSMCTNASDILLVEVFQKEMKKGTQRKTQRV 595

Zm LVKLDIRQESERHTDVIDAITTHLGIG-SYREWSEDKRQEWLLSELRGKRPLLPPDLPQTEEIA----DVIGAFHVLAELPPDSFGPYIISMATAPSDVLAVELLQRECGV---RQPLPV 561

Ft LVKLDIRQESDRHTEVLDAITQHLGIG-SYREWSEEKRQEWLLAELSGKRPLIGPDLPKTEEVK----DCLDTFKVLAELPSDCFGAYIISMATSTSDVLAVELLQREYHI---KHPLRV 555

Fp LVKLDIRQESDRHTDVLDAITQHLEIG-SYREWSEEKRQEWLLAELSGKRPLFGSDLPKTEEVK----DVLDTFNVLAELPSDCFGAYIISMATSPSDVLAVELLQRECHV---KHPLRV 556

At LVRLDIRQESERHTDVLDAITKHLDIGSSYRDWSEEGRQEWLLAELSGKRPLFGPDLPKTEEIS----DVLDTFKVISELPSDCFGAYIISMATSPSDVLAVELLQRECHV---KNPLRV 557

Ec LVRIDIRQESTRHTEALGELTRYLGIG-DYESWSEADKQAFLIRELNSKRPLLPRNWQPSAETR----EVLDTCQVIAEAPQGSIAAYVISMAKTPSDVLAVHLLLKEAGI---GFAMPV 501

*:::****** :* ..:. : * : .* .* : :* * .:***: : : : : : .* :: ..:..*::**.. .**:* *.:: :* . *

Pf VPLLETIQSLQNSSIILENLLKNPWYRKHLTTNFEDKQEIMIGYSDSGKDGGRLTSAWELFKAQEKLVHIGKKYSVEIRFFHGRGGSVS**R**GGGPQHLAILSQPINTIKNYLRVTIQGEVI 715

Zm VPLFERLAYLQSAPASVERLFSVDWYMDRI----KGKQQVMVGYSDSGKDAGRLSAAWQLYRAQEEMAQVAKRYGVKLTLFHGRGGTVG**R**GGGPTHLAILSQPPDTINGSIRVTVQGEVI 677

Ft VPLFEKLADLEAAPAAMTRLFSMDWYRNRI----DGKQEVMIGYSDSGKDAGRFSAAWQLYKTQEQIVKIAKEFGVKLVIFHGRGGTVG**R**GGGPTHLALLSQPPDTINGSLRVTVQGEVI 671

Fp VPLFEKLADLEAAPAAMARLFSIDWYRNRI----DGKQEVMIGYSDSGKDAGRFSAAWQLYKAQEEIIKVAKEFGVKLVIFHGRGGTVG**R**GGGPTHLAILSQPPDTIHGSLRVTVQGEVI 672

At VPLFEKLADLEAAPAAVARLFSIDWYKNRI----NGKQEVMIGYSDSGKDAGRLSAAWELYKAQEELVKVAKKYGVKLTMFHGRGGTVG**R**GGGPTHLAILSQPPDTVNGSLRVTVQGEVI 673

Ec APLFETLDDLNNANDVMTQLLNIDWYRGLI----QGKQMVMIGYSDSAKDAGVMAASWAQYQAQDALIKTCEKAGIELTLFHGRGGSIG**R**GGAPAHAALLSQPPGSLKGGLRVTEQGEMI 617

.**:* : *: : : .*:. ** : ..** :*:*****.**.* ::::* :::*: : : :. .::: :******::.***.* * *:**** .:::. :*** ***:*

**LOOP I**

Pf TQDFCLKGMALRSVEIYMSALLKCSLLKNTLVIKKEWRDLMDEISEISTKEYRKVVYENKDFVKYFRCATPEIEIGKLNLGSRPSKRK-EGNVESLRAIPWVF**S**WTQNRMHLSVWLGIEK 834

Zm EFCFGEEHLCFQTLQRFTAATLEHGMH-PPVSPKPEWRKLMDEMAVVATEEYRSVVVKEARFVEYFRSATPETEYGRMNIGSRPAKRRPGGGITTLRAIPWIF**S**WTQTRFHLPVWLGVGA 796

Ft EQSFGEEHLCFRTLQRFCAATLEHGMN-PPISPRPEWRELMDQMAVVATEEYRSVVFKEPRFVEYFRLATPELEFGRMNIGSRPSKRKPSGGIESLRAIPWIF**S**WTQTRFHLPVWLGFGA 790

Fp EQSFGEEHLCFRTLQRFCAATLEHGMN-PPISPRPEWRELMDQMAVVATEEYRSIVFKEPRFVEYFRLATPELEYGRMNIGSRPSKRKPSGGIESLRAIPWIF**A**WTQTRFHLPVWLGFGA 791

At EQSFGEAHLCFRTLQRFTAATLEHGMN-PPISPKPEWRALLDEMAVVATEEYRSVVFQEPRFVEYFRLATPELEYGRMNIGSRPSKRKPSGGIESLRAIPWIF**A**WTQTRFHLPVWLGFGA 792

Ec RFKYGLPEITVSSLSLYTGAILEANLL-PPPEPKESWRRIMDELSVISCDVYRGYVRENKDFVPYFRSATPEQELGKLPLGSRPAKRRPTGGVESLRAIPWIF**A**WTQNRLMLPAWLGAGT 736

: : . ::. : .* *: .: . : .** ::*::: :: . ** * :: ** *** **** * *:: :****:**: *.: :******:*:***.*: *..***

**LOOP II**

Pf IYDYLINNN--KLHIIQDMYTHWPFCTSFFNLISMVMA**K**ASIQISQEYD-ILVPEELKYIGVLLREKLKKSMELTFLVTNEKKFCDNDQLTKRSIEC**R**TKWVTVCNLIQIQALKRLREKE 951

Zm AFKFAIDKDVRNFQVLKEMYNEWPFFRVTLDLLEMVFA**K**GDPGIAGLYDELLVAEELKPFGKQLRDKYVETQQLLLQIAGHKDILEGDPFLKQGLVL**R**NPYITTLNVFQAYTLKRIRDPN 916

Ft AFKHAIQKDSKNLQMLQEMYKTWPFFRVTIDLVEMVFA**K**GNPGIAALNDKLLVSEDLRPFGESLRANYEETKNYLLKIAGHKDLLEGDPYLKQGIRL**R**DPYITTLNVCQAYTLKRIRDPN 910

Fp AFKHAIKKDSKNLQMLQEMYKTWPFFRVTIDLVEMVFA**K**GDPGIAALNDKLLVSEDLWPFGESLRANYEETKDYLLKIAGHRDLLEGDPYLKQRIRL**R**DSYITTLNVCQAYTLKRIRDPN 911

At AFRYAIKKDVRNLHMLQDMYKQWPFFRVTIDLIEMVFA**K**GDPGIAALYDKLLVSEDLWAFGEKLRANFDETKNLVLQTAGHKDLLEGDPYLKQRLRL**R**DSYITTLNVCQAYTLKRIRDAN 912

Ec ALQKVVEDG-K-QSELEAMCRDWPFFSTRLGMLEMVFA**K**ADLWLAEYYDQRLVDKALWPLGKELRNLQEEDIKVVLAIANDSHLMADLPWIAESIQL**R**NIYTDPLNVLQAELLHRSRQAE 757

:... :: * *** :.::.**:**.. :: * ** : * :* ** : . : :.. .: . . : * : *: * *:* *: :

Pf SKQKNENKDQLDNKDNSSYTNITHNYKIYENTLVTPTSEYTNVENIKQDNNQLNNSMENHNINDLNTLKINYTENYNFNKQNELFNNDEGKHMESINKNSTVRINSYLSRKKKKLIKNPK 1071

Zm FKVT-------------------------------------------------------------------------------------------------------------------- 920

Ft YHVT-------------------------------------------------------------------------------------------------------------------- 914

Fp YHVT-------------------------------------------------------------------------------------------------------------------- 915

At YNVT-------------------------------------------------------------------------------------------------------------------- 916

Ec KE---------------------------------------------------------------------------------------------------------------------- 759

.

Pf FHPLIELHPYVFPQSETENESFFDYVKTIDSNFERIPSRNNVSTYDEATNKFIDYTSLNDALIVSIKAIAAGMQ**N**TG 1148

Zm ------PQPPLSKEFAD-ENKPA-GLVKLNPASEY-------------------PPGLEDTLILTMKGIAAGMQ**N**TG 970

Ft ------LRPHISKEYAAEPSKPADELIHLNPTSEY-------------------APGLEDTLILTMKGIAAGMQ**N**TG 966

Fp ------LRPHISKEYAAEPSKPADELIHLNPTSEY-------------------APGLEDTLILTMKGIAAGMQ**N**TG 967

At ------LRPHISKEIMQ-SSKSAQELVKLNPTSEY-------------------APGLEDTLILTMKGIAAGLQ**N**TG 968

Ec -------------------------------GQEP-------------------DPRVEQALMVTIAGIAAGMR**N**TG 883

* . ::::*:::: .****::***

**Catalytic**

**peptide**
