# Supplementary material for: Phosphoenolpyruvate Carboxylase Identified as a Key Enzyme in Erythrocytic Plasmodium falciparum Carbon Metabolism
Source: PLoS Pathog. 2014 Jan 16;10(1):e1003876. doi: 10.1371/journal.ppat.1003876 (PMC3894211; doi:10.1371/journal.ppat.1003876)
Supplement: Table S8 — Primers used in this study. Restriction sites are shown in bold letters. (DOCX) [file ppat.1003876.s014.docx]

**Table S8: Primers used in this study**

| **Primer** | **Sequence (5’ to 3’)** |
| --- | --- |
| pHH1-Δ*pepc*-s | GCGC**AGATCT** TATGTTAAACTTCTAGAAGATG |
| pHH1-Δ*pepc*-as | GCGC**CTCGAG**(TTA) GAGCACTCATATATATTTCTACCG |
| pHH1-3’*pepc*-s | GCGC**AGATCT**GAGCAATACCATGGGTTTTCTC |
| pHH1-3’*pepc*-as | CGCG**CTCGAG**TCATCCTGTGTTTTGCATTCCTGCTGC |
| pCC4-Δ*pepc*-5’-s | GCGC**CCGCGG**ATGAGTAATAATCATAACAATTTCATTTATTG |
| pCC4-Δ*pepc*-5’-as | GCGC**ACTAGT**ATTAAATGTACCTTTTAATGTTTCGTTAACTGAACC |
| pCC4-Δ*pepc*-3’-s | GCGC**CCATGG**GTTTTCTCATGGACACAAAATCGAATGCATTTATC |
| pCC4-Δ*pepc*-3’-as | GCGC**CCTAGG**TAAATTTTATAATTGTGTGTAATATTTGTATAAC |

Restriction sites are in bold. Artificial stop codon of pHH1-Δ*pepc* construct is shown in brackets.
