# Supplementary material for: Phosphoenolpyruvate Carboxylase Identified as a Key Enzyme in Erythrocytic Plasmodium falciparum Carbon Metabolism
Source: PLoS Pathog. 2014 Jan 16;10(1):e1003876. doi: 10.1371/journal.ppat.1003876 (PMC3894211; doi:10.1371/journal.ppat.1003876)
Supplement: Table S9 — Chromatography conditions LC-MS. The detailed profile of the solvents used for the chromatography elution. (DOCX) [file ppat.1003876.s015.docx]

**Table S9: Chromatography conditions LC-MS**

| **Solvent A (%)** | **Solvent B (%)** | **Time (min)** |
| --- | --- | --- |
| 20 | 80 | 0 |
| 80 | 20 | 30 |
| 95 | 5 | 31 |
| 95 | 5 | 35 |
| 20 | 80 | 36 |
| 20 | 80 | 46 |
