# Supplementary material for: Phosphoenolpyruvate Carboxylase Identified as a Key Enzyme in Erythrocytic Plasmodium falciparum Carbon Metabolism
Source: PLoS Pathog. 2014 Jan 16;10(1):e1003876. doi: 10.1371/journal.ppat.1003876 (PMC3894211; doi:10.1371/journal.ppat.1003876)
Supplement: Text S1 — Supporting materials and methods and references. (DOCX) [file ppat.1003876.s016.docx]

**Supporting information – Materials and Methods**

**Cloning of pHH1 constructs.** The *P. falciparum* transfection plasmid pHH1 (Crabb et al., 2004) was used to generate a knockout construct, which disrupts the *pepc* gene by single cross-over recombination, and a control construct, which replaces the 3’ region of the *pepc* gene. The knockout fragment and the 3’ region of the *P. falciparum pepc* gene were amplified from *P. falciparum* 3D7 or D10 genomic DNA using *Pfx* Supermix (Invitrogen). Specific oligonucleotide primers were used to amplify the 1113 bp insert, equivalent to nucleotides 1094 – 2207 of the *pepc* open reading frame, producing the *pepc* knockout construct pHH1-Δ*pepc* (Table S5). The control construct pHH1-3’*pepc* encompasses the last 993 bp of the 3447 bp *pepc* gene and was amplified using primers specified in Table S8. The PCR products were subcloned into the TOPO-Blunt PCR cloning vector (Invitrogen) and the sequences were verified (Eurofins MWG Operon) before they were cloned into pHH1. Selection of transfectants was achieved using 5 nM WR99210 for parasites transfected with pHH1-Δ*pepc* and pHH1-3’*pepc.*

**Pulsed field gel electrophoresis.** To separate chromosomes, pulsed field gel electrophoresis was performed using the CHEF-DR III Variable Angle System (BioRad). Parasites were released from erythrocytes by saponin lysis, embedded in agarose blocks and incubated for 48 h at 37 ˚C in 10 mM Tris pH 8.0, 0.5 M EDTA, 1 % (v/v) sarkosyl, 2 mg/ml proteinase K. Chromosomes were separated on a 1 % (w/v) agarose gel in 1x TAE using the following parameters: 360-800 s pulse, 3 V/cm^2^ (100 Volts) for 96 h, which are optimal to separate chromosomes 11 to 14. The gels were blotted and probed with *pepc*-specific and *hDHFR* probes as described above.

**Western blotting.** Protein extracts were prepared by resuspending saponin-isolated parasite pellets in lysis buffer (PBS, 0.5 % (v/v) Triton X-100, 1 mM phenylmethylsulphonyl fluoride, 1 mM benzamidine, 20 μM leupeptin, 10 μM E64, 2 μM 1,10-phenanthroline, 4 μM pepstatin A), three cycles of freeze/thawing, followed by 5 min centrifugation at 17,000 *g* at 4 °C. Concentration of soluble protein was determined using the Bradford assay with bovine serum albumin as a standard (Bradford, 1976). Ten μg of protein from D10 and D10^Δ^*^pepc^* parasites were separated on 10 % SDS-PAGE gel and blotted onto nitrocellulose using Transblot Semidry transfer system (BioRad). The membranes were blocked in 5 % (w/v) skimmed milk powder in PBS at 4 ºC overnight before they were probed with the primary antibodies raised against *P. falciparum* MDH (1:10,000) or *P. falciparum* AAT (1:5,000) in 1 % BSA in PBS, 0.5 % TWEEN. Secondary anti-rabbit (MDH) or anti-mouse (AAT) HRP-conjugated antibodies were used at 1:10,000 dilution and the signals were visualized using the Immobilon Western kit (Millipore). A rabbit antibody against *P. falciparum* 2Cys-peroxiredoxin (Akerman and Müller, 2003) (1:100,000) was used as loading control and expression relative to the loading control was analysed using LabImage 1D software (Kapelan Bio-Imaging Solutions, Germany). The average expression of the proteins was determined from 3 to 4 independent protein extracts. Densiotometry of protein expression was analysed using LabImage 1D software (Kapelan Bio-Imaging Solutions, Germany).

**Fluorescent microscopy.** D10 and D10^Δpepc^ (maintained in routine medium for 9 days) at 8 - 10 % parasitaemia were incubated for 20 min at 37 °C with 25 nM MitoTracker Red CMXRos and for the final 5 min with 100 μg/ml Hoechst 33258 (both Invitrogen, UK), washed and resuspended in Earle’s balanced salt solution supplemented with 11 mM glucose. To collapse the mitochondrial membrane potential, the parasites were treated for 1 h with 500 nM valinomycin (Invitrogen, UK) prior to incubation with the fluorophores. Cells were viewed with an Applied Precision Deltavision Deconvolution microscope system (Olympus IX-70 invert microscope) fitted with a Coolsnap HQ camera and images were processed with the SoftWoRx software.

**Liquid chromatography - mass spectrometry (LC-MS).** The mass spectrometer was calibrated before sample analysis according to the manufacturer’s specifications. Observed mass deviations for identified compounds and standards were less than 1 ppm for both positive and negative mode. The spray needle and heated capillary line were frequently cleaned to ensure the overall pressure through the HPLC system and the foreline pressure were consistent and reproducible for each run. The following electrospray ionisation settings were used for the positive mode ionisation: Source voltage +4.00 kV (positive mode), sheath gas flow rate 57 AU, auxiliary gas flow rate 17 AU, capillary temperature 275^o^C, capillary voltage 55.00 V, tube lens voltage 100.00 V, skimmer voltage 16 V. The following electrospray settings were used for the negative mode ionisation: Source voltage -3.00 kV (negative mode), sheath gas flow rate 57 AU, auxiliary gas flow rate 17 AU, capillary temperature 275^o^C, capillary voltage -50.00 V, tube lens voltage -110.00 V, skimmer voltage -34 V. At the beginning of each sample set analysis, 2 column blanks and analytical standards for all metabolites of interest were run to ensure the system stability and confirm the identity of the metabolites of interest. Samples were loaded at random into the autosampler with the tray held at 4^o^C throughout the analytical run. 10 µL of the metabolite extract was injected onto the column. The chromatography elution protocol used is detailed in Table S9.

**Data analyses and detection of heavy isotope labelled metabolites.** The algorithm for the detection of isotope labelling begins by first determining the number of possible labelled isotopomers a metabolite can have according to its molecular formula (e.g. C_3_H_6_O_3_ could have 1, 2 or 3 carbons labelled). Then, the expected mass of each labelled isotopomer of a metabolite is calculated based on the known mass difference between light and heavy isotopes. Peaks are assigned as isotopomers if their mass is within 3 ppm of the predicted mass and within a retention time (RT) window of +/-0.2 minutes from the unlabelled peak (as isotopomers are expected to co-elute). In this study, manually curated precise RT windows were used for final peak extraction. This procedure was repeated for every possible isotopomer for each metabolite of interest to provide a comprehensive list of relative isotopomer peak areas. Isotopomer signals that were missed in the initial processing, e.g. because of low intensities or irregular peak shapes, were gap-filled in a targeted manner using mzMatch-ISO. mzMatch-ISO generates PDF files containing several plots for each metabolite: the raw chromatograms representing the monoisotopic and corresponding isotopic peaks; a normalized plot showing the variability in labelling between replicates; a trend plot of the pattern of labelling in each sample group; and a plot quantifying the absolute labelling pattern of a selected isotopomer of interest. mzMatch-ISO can also distinguish natural abundance for each metabolite versus the isotopomer where only one carbon is labelled (this is especially useful to distinguish bicarbonate labelling). Such plots and the associated data were collated to generate a comprehensive view of labelling profile for each metabolite. This process and how the data are presented are shown schematically in Figure S6.

**Statistical analyses.** Experiments were performed normally at least in triplicate using independent parasite cultures or preparations. Graphs were generated and statistical analyses performed using GraphPad Prism version 5 for Windows (GraphPad Software, San Diego California USA). Significant differences in growth of the D10^Δ^*^pepc^* clones in supplemented media, compared to complete medium, was tested using ANOVA with the Dunnett’s Multiple Comparison post-test. The IC_50_ values were calculated by nonlinear regression of the sigmoidal dose-response equation (GraFit Software) and significance tested using ANOVA with the Newman Keuls post-test.

**Gene IDs and NCBI Accession numbers.** The genes/proteins referred to in the paper have the following identification numbers and have been identified with PlasmoDB (<http://plasmodb.org/plasmo/>) for the *Plasmodium* genes or the NCBI accession number for the human genes: *P. falciparum* aspartate transaminase: PF3D7_0204500; *P. falciparum* carbamoyl phosphate synthetase: PF3D7_1308200; *P. falciparum* carbonic anhydrase: PF3D7_1140000; *P. falciparum* dicarboxylate-tricarboxylate carrier: PF3D7_0823900; *P. falciparum* dihydroorotate dehydrogenase: PF3D7_0603300; FAD-dependent glycerol 3-phosphate dehydrogenase: PF3D7_0306400; *P. falciparum* fumarate hydratase: PF3D7_0927300; *P. falciparum* glycerol 3-phosphate dehydrogenase 2: PF3D7_1216200; *P. falciparum* malate dehydrogenase: PF3D7_618500; *P. falciparum* malate:quinone oxidoreductase: PF3D7_0616800; *P. falciparum* phosph*enol*pyruvate carboxylase: PF3D7_1426700; *P. falciparum* phosph*enol*pyruvate carboxykinase: PF3D7_1342800; *P. falciparum* pyruvate dehydrogenase E1α: PF3D&_1124500; *P. falciparum* pyruvate dehydrogenase E1β: PF3D&_1446400; *P. falciparum* pyruvate dehydrogenase E2: PF3D7_1020800; *P. falciparum* pyruvate dehydrogenase E3: PF3D7_0815900 *P. falciparum* 2Cys-peroxiredoxin: PF3D7_1438900. The NCBI accession number for the human proteins are: *H. sapiens* argininosuccinate lyase: AAA51788.1; *H. sapiens* argininosuccinate synthase: NP_000041.2; *H. sapiens* aspartate:glutamate carrier: O75746.2; *H. sapiens* fumarate hydratase: AAP88841.1; *H. sapiens* NADP^+^-dependent malic enzyme: NP_001155058.1; *H. sapiens* mitochondrial aspartate aminotransferase: AAH00525.1. The accession number of the *pepc* genes used in the multiple alignment are: *Arabidopsis* *thaliana* phosphoenolpyruvate carboxylase 3: NP_188112;  *Escherichia* *coli* PEPC (B7MR83); *Flaveria* *pringlei* PEPC (Q01647); *F.* *trinervia* (P30694); Pf, *P.* *falciparum* PEPC (PF3D7_1426700); *Zea* *mays* PEPC (ACJ38542).

**Supplemental References**

Akerman, S.E., and Müller, S. (2003). 2-Cys peroxiredoxin PfTrx-Px1 is involved in the antioxidant defence of Plasmodium falciparum. Mol. Biochem. Parasitol. *130*, 75-81.

Bradford, M.M. (1976). A rapid and sensitive method for the quantitation of microgram quantities of protein utilizing the principle of protein-dye binding. Anal. Biochem *72*, 248-254.

Crabb, B.S., Rug, M., Gilberger, T.W., Thompson, J.K., Triglia, T., Maier, A.g., and Cowman, A.F. (2004). Transfection of the human malaria parasite Plasmodium falciparum. Methods Mol. Biol. *270*, 263-276.
